# Supplementary material for: PhyloFisher: A phylogenomic package for resolving eukaryotic relationships
Source: PLoS Biol. 2021 Aug 6;19(8):e3001365. doi: 10.1371/journal.pbio.3001365 (PMC8345874; doi:10.1371/journal.pbio.3001365)
Supplement: S1 Text — Table A: Taxonomic composition of the PhyloFisher v. 1.0 dataset. Fig A: Phylogenomic tree of 304 taxa, 240 orthologs, and 72,632 amino acid sites (gt80 matrix). Supermatrix was processed as described above in the matrix_constructor.py methodology. The tree was built using IQ-TREE under LG+G4+F+C60+PMSF with an LG+G4+F+C20 input tree for generation of the PMSF site frequencies inferred in IQ-TREE with 350 real bootstrap replicates (MLBS). This is the uncollapsed version of the tree shown in Fig 3 of the main text (with the branches and nodes colored in the same way). MLBS values of 100% are not shown; all other values are indicated at their respective node. Data associated with this figure are available in the directory archive FigA.tgz within the data archive available from https://ir.library.msstate.edu/bitstream/handle/11668/19731/Tice_etal.PhyloFisher.DATA.tar.gz. Fig B: Violin plot of each gene’s RTC score per method of trimming and untrimmed. Quartiles are drawn on the violin plots, overlaid with box and whisker plots. Data associated with this figure are available in the directory archive FigB.tgz within the data archive available from https://ir.library.msstate.edu/bitstream/handle/11668/19731/Tice_etal.PhyloFisher.DATA.tar.gz. Fig C: Box and whisker plot of the pairwise difference between each gene’s RTC score per method of trimming to the untrimmed RTC score. Data associated with this figure are available in the directory archive FigC.tgz within the data archive available from https://ir.library.msstate.edu/bitstream/handle/11668/19731/Tice_etal.PhyloFisher.DATA.tar.gz. Fig D: Violin plot of each node in the ML tree’s gene concordance factor assessed through IQ-TREE per method of trimming. Quartiles are drawn on the violin plots, overlaid with box and whisker plots. Data associated with this figure are available in the directory archive FigD.tgz within the data archive available from https://ir.library.msstate.edu/bitstream/handle/11668/19731/Tice_etal.Phy [file pbio.3001365.s002.docx]

***PLoS Biology* - Methods and Resources Article**

**PhyloFisher: A phylogenomic package for resolving eukaryotic relationships**

**Supplemental Materials and Methods**

**Software packages used in the PhyloFisher package**

*If you use PhyloFisher please also cite these articles*

ASTRAL-III (Zhang et al., 2018) - Zhang C, Rabiee M, Sayyari E, Mirarab S. ASTRAL-III: polynomial time species tree reconstruction from partially resolved gene trees. *BMC Bioinformatics*. 2018;19: 153. doi:10.1186/s12859-018-2129-y

BLAST v. 2.9.0 (Camacho et al., 2009) - Camacho, C., Coulouris, G., Avagyan, V., Ma, N., Papadopoulos, J., Bealer, K., & Madden, T. L. (2009). BLAST+: Architecture and applications. *BMC Bioinformatics*, *10*(1), 421. https://doi.org/10.1186/1471-2105-10-421

BMGE v. 1.1.2 (Criscuolo & Gribaldo, 2010) - Criscuolo, A., & Gribaldo, S. (2010). BMGE (Block Mapping and Gathering with Entropy): A new software for selection of phylogenetic informative regions from multiple sequence alignments. *BMC Evolutionary Biology*, *10*(1), 210. https://doi.org/10.1186/1471-2148-10-210

CD-HIT v. 4.8.1 (Fu et al., 2012) - Fu, L., Niu, B., Zhu, Z., Wu, S., & Li, W. (2012). CD-HIT: accelerated for clustering the next-generation sequencing data. *Bioinformatics*, *28*(23), 3150–3152. https://doi.org/10.1093/bioinformatics/bts565

DIAMOND v. 09.24 (Buchfink et al., 2015) - Buchfink, B., Xie, C., & Huson, D. H. (2015). Fast and sensitive protein alignment using DIAMOND. *Nature Methods*, *12*(1), 59–60. https://doi.org/10.1038/nmeth.3176

DIST_EST v.1.0 (Susko et al., 2003) - Susko, E., & Roger, A. J. (2007). On Reduced Amino Acid Alphabets for Phylogenetic Inference. *Molecular Biology and Evolution*, *24*(9), 2139–2150. https://doi.org/10.1093/molbev/msm144

DIVVIER v. 1.01 (Ali et al., 2019) - Ali, R. H., Bogusz, M., & Whelan, S. (2019). Identifying Clusters of High Confidence Homologies in Multiple Sequence Alignments. *Molecular Biology and Evolution*, *36*(10), 2340–2351. https://doi.org/10.1093/molbev/msz142

ETE3 3.1.1 (Huerta-Cepas et al., 2016) - Huerta-Cepas, J., Serra, F., & Bork, P. (2016). ETE 3: Reconstruction, Analysis, and Visualization of Phylogenomic Data. *Molecular Biology and Evolution*, *33*(6), 1635–1638. https://doi.org/10.1093/molbev/msw046

FastTree v. 2.1.11 (Price et al., 2010) - Price, M. N., Dehal, P. S., & Arkin, A. P. (2010). FastTree 2—Approximately Maximum-Likelihood Trees for Large Alignments. *PLoS ONE*, *5*(3), 1–10. a9h.

HMMER v. 3.2.1 (Mistry et al., 2013) - Mistry, J., Finn, R. D., Eddy, S. R., Bateman, A., & Punta, M. (2013). Challenges in homology search: HMMER3 and convergent evolution of coiled-coil regions. *Nucleic Acids Research*, *41*(12), e121–e121. https://doi.org/10.1093/nar/gkt263

MAFFT v.7.455 (Katoh & Standley, 2013) - Katoh, K., & Standley, D. M. (2013). MAFFT Multiple Sequence Alignment Software Version 7: Improvements in Performance and Usability. *Molecular Biology and Evolution*, *30*(4), 772–780. https://doi.org/10.1093/molbev/mst010

MAMMaL v1.1.1 (Susko et al., 2018) - Susko, E., Lincker, L. and Roger, A.J. (2018). Accelerated Estimation of Frequency Classes in Site-heterogeneous Profile Mixture Models. *Molecular Biology and Evolution*. 9:1266-1283.

OrthoMCL v 5.0 (Chen et al., 2006) - Chen, F., Mackey, A. J., Stoeckert, C. J., Jr, & Roos, D. S. (2006). OrthoMCL-DB: querying a comprehensive multi-species collection of ortholog groups. *Nucleic Acids Research*, *34*(suppl_1), D363–D368. https://doi.org/10.1093/nar/gkj123

PREQUAL v. 1.02 (Whelan et al., 2018) - Whelan, S., Irisarri, I., & Burki, F. (2018). PREQUAL: detecting non-homologous characters in sets of unaligned homologous sequences. *Bioinformatics*, *34*(22), 3929–3930. https://doi.org/10.1093/bioinformatics/bty448

RAxML v. 8.2.12 (Stamatakis, 2014) - Stamatakis, A. (2014). RAxML version 8: A tool for phylogenetic analysis and post-analysis of large phylogenies. *Bioinformatics*, *30*(9), 1312–1313. https://doi.org/10.1093/bioinformatics/btu033

trimAl v.1.4.rev15 (Capella-Gutiérrez et al., 2009) - Capella-Gutiérrez, S., Silla-Martínez, J. M., & Gabaldón, T. (2009). trimAl: A tool for automated alignment trimming in large-scale phylogenetic analyses. *Bioinformatics*, *25*(15), 1972–1973. https://doi.org/10.1093/bioinformatics/btp348

**Table A. Taxonomic Composition of the PhyloFisher v. 1.0 dataset**

| **Taxonomic Group** | **Number of Taxa** |
| --- | --- |
| Stramenopiles | 60 |
| Obazoa | 49 |
| Amoebozoa | 42 |
| Alveolata | 27 |
| Discoba | 19 |
| Rhizaria | 18 |
| Chloroplastida | 17 |
| Rhodophyta | 15 |
| Metamonada | 10 |
| Haptophyta | 10 |
| Cryptista | 9 |
| CRuMs | 4 |
| Glaucophyta | 4 |
| Ancyromonada | 4 |
| Centroheliozoa | 4 |
| Malawimonada | 3 |
| Telonemia | 3 |
| Hemimastigophora | 2 |
| Rhodelphidia | 2 |
| Picozoa | 1 |
| *Ancoracysta* | 1 |
| **TOTAL** | **304** |

**
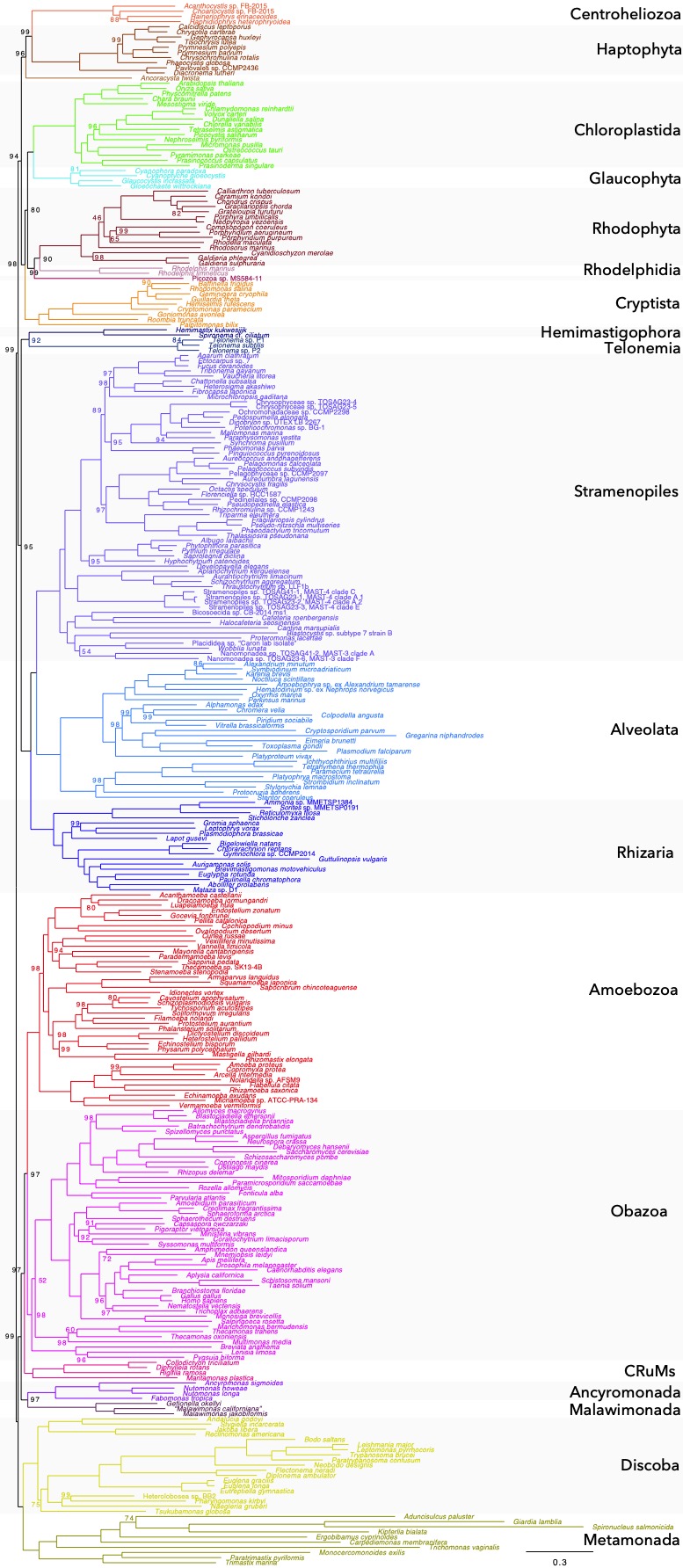
**

**Fig A** Phylogenomic tree of 304 taxa, 240 orthologs and 72,632 amino acid sites (gt80 matrix). Supermatrix was processed as described above in the *matrix_constructor.py* methodology. The tree was built using IQ-TREE under LG+G4+F+C60+PMSF with an LG+G4+F+C20 input tree for generation of the PMSF site frequencies inferred in IQ-TREE with 350 real bootstrap replicates (MLBS). This is the uncollapsed version of the tree shown in Fig 3 of the main text (with the branches and nodes colored in the same way). MLBS values of 100% are not shown, all other values are indicated at their respective node. Data associated with this figure is available in the directory archive FigA.tgz within the data archive available from https://ir.library.msstate.edu/bitstream/handle/11668/19731/Tice_etal.PhyloFisher.DATA.tgz.

**Trimming experiments**

In order to examine and understand the implication of using various trimming methods on our resulting phylogenomic analyses, we conducted a series of experiments to provide end-users with a reasonable default approach to processing single orthologs into a phylogenomic concatenated supermatrix (via *matrix_constructor.py*). While we recognize that there are other parameters and programs that may be used by experienced users, it is our goal to provide a rationalized methodology for users of our package that 1) provides reproducible results, 2) is not too computationally demanding in terms of processing the input data and the output matrix, and 3) uses state-of-the-art methodology. Our rationale for using a gap-threshold of 80% (i.e., using a trimming method that is based solely on site-occupancy of alignments) in trimAl is to reduce the number of sites to a reasonably sized matrix without negating phylogenetic signal. As our dataset consists of 304 taxa and 240 orthologs, the number of phylogenetically useable sites and overall matrix size is quite large, leading to potentially unattainable computational resource requirements for many end-users.

**Single-protein tree relative tree certainty score per trimming method.** Alignments were processed according to the methods within the PhyloFisher workflow. That is, each ortholog fasta file was collected and run through PREQUAL, MAFFT-GINSI, DIVVIER (Partial) and then trimmed. In order to test the impact of various trimming methods on individual single-protein alignments, trees were computed from trimmed alignments generated by BMGE (global entropy of 0.1, 0.3, 0.6 and 0.9) and trimAl (Gap Threshold of 1%, 50%, and 80%). From these single-protein trees inferred from the individual trimming methods, Relative Tree Certainty (RTC) Scores were computed in RAxML from the best tree and bootstrap trees inferred under PROTCATLGF model using rapid 100 rapid bootstraps. Alignments were also processed with no trimming following the PREQUAL, MAFFT-GINSI, and DIVVIER process, and trees were inferred under the same methodology. For RTC scores, higher values equate to a higher confidence in the alignment to yield the tree. Each resultant RTC score was assessed against each method. Additionally, the best RTC score was also collected and is plotted along with the RTC scores of gene trees inferred using the above methods (Fig B,C). The highest mean RTC is for trimAl with a gt of 1%. For the BestRTC score, trimAl gt1 is selected as best 49% of the time.

**Fig B** Violin Plot of each gene’s RTC score per method of trimming and untrimmed. Quartiles are drawn on the violin plots, overlaid with box and whisker plots. Data associated with this figure is available in the directory archive FigB.tgz within the data archive available from https://ir.library.msstate.edu/bitstream/handle/11668/19731/Tice_etal.PhyloFisher.DATA.tgz.

data <- read.csv("/Users/mbrown/Documents/MyDocuments-Aine/MyManuscripts-AINE/Phylofisher1.0.Manuscript/METHODS/RTC-scores.dataframed-BMGEseries.csv")

p <- ggplot(data, aes(factor(Method), RTC))

p + geom_violin(scale = "count",draw_quantiles = c(0.25, 0.5, 0.75), aes(fill = Method)) + geom_boxplot(width=.2)

**Fig C** Box and whisker plot of the pairwise difference between each gene’s RTC score per method of trimming to the untrimmed RTC score. Data associated with this figure is available in the directory archive FigC.tgz within the data archive available from https://ir.library.msstate.edu/bitstream/handle/11668/19731/Tice_etal.PhyloFisher.DATA.tgz.

> data <- read.csv("/Users/mbrown/Documents/MyDocuments-Aine/MyManuscripts-AINE/Phylofisher1.0.Manuscript/METHODS/RTC-TrimalBMGEseries-PAIRWISE.csv")

> p <- ggplot(data, aes(factor(Method), Pairwise))

> p + geom_boxplot(draw_quantiles = c(0.25, 0.5, 0.75), aes(fill = PAIR) + geom_boxplot(width=2))

**Gene concordance factor per single-protein alignment trimming method.** Finally, to assess the concordance between trimming methods of orthologs and the global phylogeny inferred through each phylogenomic matrix constructed, we used the Gene Concordance Factor in IQ-TREE 2 [1], under LG+G4+F+C60+PMSF with an LG+G4+F+C20 input tree for site frequencies for PMSF. Matrices assessed in this fashion were the BMGE3, trimAl GT1, trimAl GT50, and trimAl GT80 trimming methodologies. The gCF values were calculated using the overall global ML tree based on the trimming method with bootstrap tree of each individual gene trimmed using the same method as used for the supermatrix. Each gCF score per the node within the ML tree was plotted in R as a violin plot. The highest mean is trimAl with a gt of 1% (Fig D).

**Fig D** Violin Plot of each node in the ML tree’s gene concordance factor assessed through IQ-TREE per method of trimming. Quartiles are drawn on the violin plots, overlaid with box and whisker plots. Data associated with this figure is available in the directory archive FigD.tgz within the data archive available from https://ir.library.msstate.edu/bitstream/handle/11668/19731/Tice_etal.PhyloFisher.DATA.tgz.

iqtree2 -t public_forged_1.17.20.LGGF.PMSF.treefile --gcf gt1trimal.RAxMLbootstrapTrees.txt --prefix concord.gCF.BS.gt1trimal

iqtree2 -t public.gt50trimal.out.LGGF.PMSF1.treefile --gcf gt50trimal.RAxMLbootstrapTrees.txt --prefix concord.gCF.BS.gt50trimal

iqtree2 -t public.gt80trimal.out.LGGF.PMSF1.treefile --gcf gt80trimal.RAxMLbootstrapTrees.txt --prefix concord.gCF.BS.gt80trimal

iqtree2 -t public.bmge.out.LGGF.PMSF1.treefile --gcf bmge.RAxMLbootstrapTrees.txt --prefix concord.gCF.BS.bmge

> data <- read.csv("/Users/mbrown/Documents/MyDocuments-Aine/MyManuscripts-AINE/Phylofisher1.0.Manuscript/METHODS/GCF-Concordance/GeneConcordance.csv")

> p <- ggplot(data, aes(factor(Method), gCF))

> p + geom_violin(scale = "count",draw_quantiles = c(0.25, 0.5, 0.75), aes(fill = Method)) + geom_boxplot(width=.2)

**Impact of trimming on phylogenomic tree node bootstrap values.** To test the impact of the above trimming methods on the global phylogenomic tree, individual alignments were processed as above with BMGE (global entropy of 0.3) and trimAl with a Gap threshold (gt) of 1%, 50%, and 80%. Additionally, the highest scoring individual RTC scored alignment for each gene was also collected. Phylogenomic matrices were constructed using PhyloFisher’s *matrix_constructor.py*. This resulted in 4 total phylogenomic supermatrices that were further investigated, i.e., BMGE3, trimAl GT1, trimAl GT50, and trimAl GT80. Trees from each supermatrix were inferred using the following methodology, first a ML tree was inferred in IQ-TREE under the LG+G4+C20+F model. This tree was then used (IQ-TREE option -ft) to compute individual Posterior Mean Site Frequencies (PMSF) under the LG+G4+C60 model and an ML tree with 1000 ultrafast bootstrap (BS) replicates was inferred. Nodes of particular interest in the bootstrap trees were collected as groups with the *bipartition_examiner.py* script of the PhyloFisher package (“*bipartition_examiner.py -g groups.txt -b ufbootlist.txt”* where -g is the groups of interest constructed as such “GROUP1: TaxonA, TaxonB, TaxonC” and -b is a list of bootstrap tree files). Bootstrap values of bipartitions of interest were plotted. We see that the phylogeny (and bootstrap support) is independent on the trimming method used when using the above approach (Fig E). However, it should be noted that often in phylogenomic analyses users will run a simple site homogeneous model (LG+G4+F), which is computationally inexpensive, as an input tree to infer PMSF site frequencies for the LG+G4+F+C60-PMSF model (example of this is [2] and [3], as well as many others). Interestingly, we found that when a site homogenous model was used as an input guide tree for the site frequency calculation for PMSF under the LG+G4+F+C60-PMSF site heterogeneous model, we see that conflicting topologies are obtained, which are often highly supported (Fig F).

**Fig E** Bootstrap values of nodes of interest, inferred in IQ-TREE under LG+G4+F+C60+PMSF with an LG+G4+F+C20 input tree for generation of the PMSF site frequencies inferred in IQ-TREE with 1000 ultrafast bootstrap replicates (MLBS). This analysis highlights that different alignment trimming methods have little effect on the output tree and the bootstrap support values when a site heterogeneous model is used. Data associated with this figure is available in the directory archive FigE.tgz within the data archive available from https://ir.library.msstate.edu/bitstream/handle/11668/19731/Tice_etal.PhyloFisher.DATA.tgz.

**

**Fig F** Bootstrap values of a few selected nodes of interest displayed in Fig 3 are illustrated here, inferred in IQ-TREE under LG+G4+F+C60+PMSF with either an LG+G4+F or an LG+G4+F+C20 input tree for generation of the PMSF site frequencies inferred in IQ-TREE with 1000 ultrafast bootstrap replicates (MLBS). Conflicting topologies with high support are found in the LG+G4+F input tree analysis, while nodes and topologies do not conflict when inferred with LG+G4+F+C20 as the input tree. Data associated with this figure is available in the directory archive FigF.tgz within the data archive available from https://ir.library.msstate.edu/bitstream/handle/11668/19731/Tice_etal.PhyloFisher.DATA.tgz.

**Overall trimming results*.*** While in most cases, no trimming or very light trimming with a gap-threshold of 1.0% generally perform better with regard to the relative tree confidence score as assessed through RAxML (Fig B and Fig C) as well as with respect to gene concordance factor assessed through IQ-TREE (Fig D). However, the overall impact of trimming method had little effect on the resultant tree and the nodal support on that tree (Fig E) when these supermatrices were assessed under LG+G4+F+C60-PMSF with an LG+G4+F+C20 input tree for generation of the PMSF site frequencies. Regardless, the computational time required is highly dependent on the number of sites in the supermatrix. The size of supermatrix varies from 102,967 to 63,969 amino acid sites (Fig G). Because the trimAl (-gt 0.80) gap threshold of 80% supermatrix has an intermediate number of sites (72,632) of the supermatrices examined, which is computationally less expensive than the gt1 matrix, and shows the same overall topology regardless of the trimming method (Fig E), we suggest that this represents an acceptable methodology to be used as a default method for *matrix_constructor.py.*

**Fig G** Histogram of amino acid sites of the supermatrices generated per each trimming method. Data associated with this figure is available in the directory archive FigG.tgz within the data archive available from https://ir.library.msstate.edu/bitstream/handle/11668/19731/Tice_etal.PhyloFisher.DATA.tgz.

**Fast site removal examination.** In large scale phylogenomic analyses, the fastest evolving sites in the supermatrix are expected to be most susceptible to phylogenetic saturation and systematic error [19]. These errors likely originate from model misspecification in large scale analyses. To examine the effect that these rapidly evolving sites have on our results, we include a utility (*fast_site_remover.py*) to calculate the rates of evolution given a supermatrix and the output phylogenomic tree (“fast_site_remover.py -m public.gt80trimal.out -tr public.gt80trimal.PMSF-c20inputtree.treefile -c 9000”). The rates of evolution at each site of the 304-taxon 240-ortholog dataset were estimated with DIST_EST [20] under the LG model using discrete gamma probability estimation. Sites were ordered based on their rate of evolution and the sites with the highest rates were removed in a stepwise fashion from the input dataset, with 9000 sites removed per step (Fig H). From this analysis, we see a marked increase in bootstrap values for the nodes of interest at 9000 sites removed. The tree from this dataset is shown in Fig I and was used for the main text Fig 3.

** Fig H** Fast site removal of sites from the whole dataset (the gt80 trimAl matrix of 72,632 amino acid sites). Each step has 9000 sites removed in a fastest to slowest stepwise manner to exhaustion. ML tree was inferred for each dataset in IQ-TREE under LG+G4+F+C60+PMSF with an LG+G4+F+C20 input tree for generation of the PMSF site frequencies inferred in IQ-TREE with 1000 ultrafast bootstrap replicates (UFBOOT). The tree from 9000 sites removed (9K) is shown in Fig I, and represents the tree shown in Fig 3 of the main text. Data associated with this figure is available in the directory archive FigH.tgz within the data archive available from https://ir.library.msstate.edu/bitstream/handle/11668/19731/Tice_etal.PhyloFisher.DATA.tgz.

**
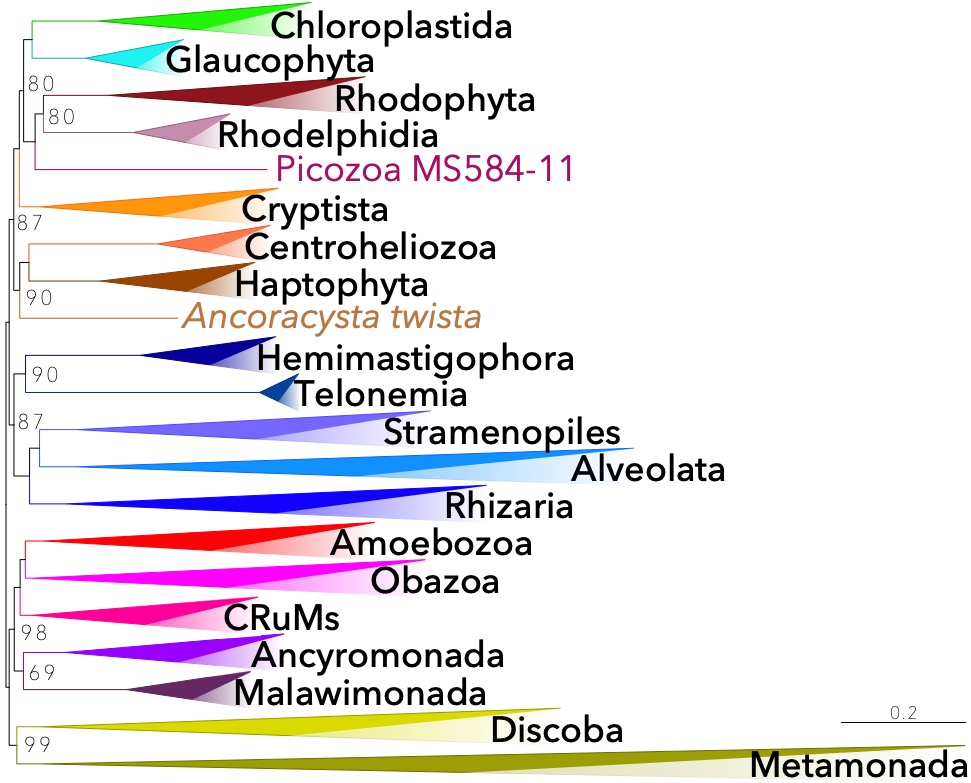
**

**Fig I** Phylogenomic cartoon tree of 304 taxa 240 orthologs and 63,632 amino acid sites, with the top 9000 of the fastest evolving sites in the original supermatrix removed (as indicated by fast_site_remover.py, see Fig H). The resulting supermatrix was processed as described above in the *matrix_constructor.py* methodology. IQ-TREE under LG+G4+F+C60+PMSF with an LG+G4+F+C20 input tree for generation of the PMSF site frequencies inferred in IQ-TREE with 200 real maximum likelihood bootstrap replicates (MLBS). Branches and nodes are colored as shown in Fig 3 of the main text. MLBS values of 100% are not shown, all other values are indicated at their respective node. Data associated with this figure is available in the directory archive FigI.tgz within the data archive available from https://ir.library.msstate.edu/bitstream/handle/11668/19731/Tice_etal.PhyloFisher.DATA.tgz.

**Random subsampling of genes.** We include a random subsampling of orthologs utility, which will subsample the user’s input ortholog datasets and construct randomly subsampled supermatrices at a user-defined percentage of sampling in a stepwise fashion. This utility calculates the number of supermatrices necessary to construct and infer using the percent subsampling (-ps) under a default, but user definable, confidence interval of 0.95 (-ci). This utility uses the formula ci=1-(1-x/100)^n to determine the number of replicates where ci is the confidence interval (i.e., 0.95), x is the percentage of genes samples and n is the number of replicates, as in [2]. The subsamples were then inferred in IQ-TREE under LG+G4+F+C60+PMSF with an LG+G4+F+C20 input tree for generation of the PMSF site frequencies inferred in IQ-TREE with 1000 ultrafast bootstrap replicates (MLBS) (Fig J).


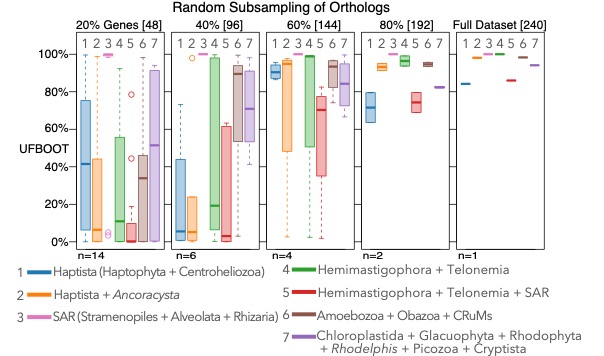


**Fig J** Random subsampling of using the *random_sample_iteration.py* utility.

(“*random_sample_iteration.py -i gt80trimal.fastas/ -f phylip-relaxed -ci 0.95 -ps 20*”). Each replicate was inferred in IQ-TREE under LG+G4+F+C60+PMSF with an LG+G4+F+C20 input tree for generation of the PMSF site frequencies inferred in IQ-TREE with 1000 ultrafast bootstrap replicates (MLBS). The support values of nodes of interest were calculated with the PhyloFisher utility *bipartition_examiner.py* and plotted in R using the boxplot function in the gplots library. Data associated with this figure is available in the directory archive FigJ.tgz within the data archive available from https://ir.library.msstate.edu/bitstream/handle/11668/19731/Tice_etal.PhyloFisher.DATA.tgz.

**Amino acid compositional bias examination.**

To examine the potential impact of compositional heterogeneity of amino acid sequence composition among taxa in phylogenomic matrices, we developed a utility *aa_comp_calculator.py*. This utility (“*aa_comp_calculator.py -i ./public.gt80trimal.out*”) calculates the amino acid compositions and creates a matrix for each taxon in our phylogenetic dataset. From this amino acid composition matrix, we used the hierarchical clustering (using the scipy.cluster.hierarchy algorithm) to group them together on the basis of Euclidean distances between their amino acid frequencies (Fig K). This utility outputs a Newick tree file as well as pdf of the clustering. Taxa that have similar amino acid compositions will tend cluster together in the hierarchical clustering irrespective of the real phylogenetic signal of the taxa.

**Fig K** Hierarchical clustering of amino acid compositions of our supermatrix. Colors are depictions of taxa as labeled in Fig 3 of the main text. Data associated with this figure is available in the directory archive FigK.tgz within the data archive available from https://ir.library.msstate.edu/bitstream/handle/11668/19731/Tice_etal.PhyloFisher.DATA.tgz.

**Heterotachious site removal examination.** Heterotachious sites are sites that are evolving at differing rates across branches in the tree (i.e., across taxa within the matrix); these are also susceptible to generate phylogenetic reconstruction artifacts, much like fast evolving sites. Indeed, these sites are not properly fitted by standard site-homogeneous models of evolution like the C-series (C20 and C60, used here); these models account for different rates amongst sites, but not within a particular site. In order to examine the effect of heterotachious sites, we developed a utility (*heterotachy.py*) that identifies and removes these sites in a stepwise fashion, constructing a supermatrix for each step. This utility was run using the command “*heterotachy.py -m public.gt80trimal.out -t public.gt80trimal.PMSF-c20inputtree.treefile*”, where *-m* is the supermatrix (72,632 dataset, gt80trimal) and -t is the IQ-TREE under LG+G4+F+C60+PMSF with an LG+G4+F+C20 input tree for generation of the PMSF site frequencies from that matrix (Figure S1). To identify heterotachy, the *heterotachy.py* utility takes a multistep approach listed here. 1) The rates of evolution of taxa in the tree are estimated using branch lengths from our full dataset supermatrix tree (Figure S1). Since we do not know *a priori* where the root of the eukaryotes lies, to get the most reliable calculation of branch-length per taxon, we calculate the average tip-to-tip branch-length between a given taxon and every other taxon in the tree. Such branch length measurement would be skewed by the fact that some taxa have more numerous and closer representatives in the tree than others (because of the bias in taxon sampling in existing sequence data), which will artificially reduce this estimated average branch-length (e.g., if you have 5 closely related taxa at the end of a very long branch, we still want to consider them long branching taxa). To counteract this, we only averaged over the 50% longest tip-to-tip branches between each given taxon and the rest. 2) We bin the taxa based on their branch lengths into thirds (Fast=long, Slow=short, Medium=medium). 3) Select taxa from the supermatrix to generate a FAST and a SLOW supermatrix, disregarding the medium rate taxa. 4) Prune the tree into a FAST and a SLOW tree only containing the taxa in each respective group (FAST or SLOW). 5) Calculate the site rates based on the pruned tree and the pruned dataset (FAST and SLOW), using DIST_EST under the LG model using discrete gamma probability estimation. 6) Calculate the ratio of site rates (between FAST to SLOW) (which will reflect whether a site evolves much faster in fast-evolving lineages than in slow-evolving ones) and rank them from highest to lowest. 7) Remove the highest to lowest ratio of computed heterotachy in a stepwise fashion from the full dataset supermatrix. A phylogenomic tree from each supermatrix was inferred in IQ-TREE under LG+G4+F+C60+PMSF with an LG+G4+F+C20 input tree for generation of the PMSF site frequencies inferred in IQ-TREE with 1000 ultrafast bootstrap replicates (MLBS) (Fig L).

**Fig L** Heterotachious site removal of sites from the whole dataset (72,632 amino acid). Step0 and Step1 have 3000 sites removed (see rationale below and Fig M) and then each subsequent step has 9000 sites removed using the greatest to least heterotachy ratio stepwise manner to exhaustion. ML tree was inferred for each dataset in IQ-TREE under LG+G4+F+C60+PMSF with an LG+G4+F+C20 input tree for generation of the PMSF site frequencies inferred in IQ-TREE with 1000 ultrafast bootstrap replicates (UFBOOT). Data associated with this figure is available in the directory archive FigL.tgz within the data archive available from https://ir.library.msstate.edu/bitstream/handle/11668/19731/Tice_etal.PhyloFisher.DATA.tgz.

To examine the expected among site rate variation given our ML tree as accounted for within the LG+G4+C60+F model of evolution, a null-distribution of fast to slow taxa site rates was inferred from simulated data under the LG+G4+C60+F model using the *heterotachy.py* utility. This distribution may be used to identify sites in our real dataset that may be interpreted as more heterotachious than expected, which are considered outliers and are removed and investigated further. To do this, a simulated dataset was generated using SiteSpecific.seq-gen (Sequence Generator - seq-gen, version 1.3.2 – recoded for use in Wang et al. 2018) for simulating data under C20 and C60 heterogeneous models of evolution. This program uses the 60 C60 profiles + the frequency profile of an amino acid exchange matrix as output from IQ-tree (iqtree -nt 12 -wbtl -m LG+C60+F+G -s public.gt80trimal.out -pre public.gt80trimal.LGc60GF -mem 160G). From this log file of IQ-TREE the mixture weights for C60 (Mixture weights: 0.072 0.019 0.005 0.013 0.030 0.032 0.009 0.006 0.009 0.008 0.045 0.013 0.011 0.008 0.014 0.008 0.005 0.022 0.028 0.007 0.004 0.001 0.032 0.012 0.021 0.013 0.020 0.025 0.028 0.050 0.045 0.008 0.009 0.022 0.024 0.021 0.020 0.006 0.013 0.003 0.016 0.005 0.008 0.014 0.015 0.012 0.001 0.016 0.010 0.005 0.016 0.008 0.034 0.006 0.024 0.023 0.005 0.005 0.013 0.016 0.006) and the gamma shape alpha (Gamma shape alpha: 0.749) were printed. These were used as input for SiteSpecific.seq-gen to generate a 100,000 amino acid site simulated dataset (SiteSpecific.seq-gen -mLG -g 4 -a 0.785 -p 61 -C60 2 -l 100000 -wt c60weights.txt public.gt80trimal.LGc60GF.treefile > SeqGenC60.phy). Using this simulated dataset and the output tree from the real data (public.gt80trimal.LGc20GF.treefile) we used PhyloFisher’s utility *heterotachy.py (“heterotachy.py -t public.gt80trimal.LGc20GF.treefile -m SeqGenC60.phy”*) to generate the site rates for fast and slow taxa as described in the *heterotachy.py* description (“*heterotachy.py -t public.gt80trimal.LGc60GF.treefile -m SeqGenC20.phy*”). Using the output site rates for Fast and Slow taxa (i.e., fast.rate_est.dat and slow.rate_est.dat), a ratio of Fast to Slow was computed for each site. These ratios were sorted and used to calculate the p-value of the outlier heterotachious sites (Fig M) for the real dataset (Figure S14). The largest ratio from the LG+G4+C60+F simulation, 9.08, represents a p-value of 1/100,000 or p-value = 0.00001 (Fig M). Therefore, a removal of all sites above a ratio of 9.08 in our real data results in a removal of 3053 sites (Fig N) (p=0.00001), however this was round to the closest 1000 sites (i.e., 3000 removed with a fast/slow ratio greater than 9.23). At 6000 sites removed from the real data, the fast/slow ratio is 3.48, which corresponds to the simulated data at ranked site 280, with a p-value equal to 280/100,000 or 0.0028 (Fig N). At 9000 sites removed from the real data, the ratio is 9.23, which corresponds to the simulated data at site 1265, with a p-value equal to 1265/100,000 or 0.0126 (Fig N).

**Fig M** Ratio of fast to slow taxa site rates, on a per site basis, estimated from a simulated dataset. This dataset was simulated under the LG+G4+C60+F model of evolution using our output tree under this model with our gt80 dataset. Fast/slow taxa site ratios were estimated using the *heterotachy.py* utility. The maximum observed ratio was 9.08 in simulated data. This set of ratios was used further as a null-distribution of expected fast/slow ratios under this model. Data associated with this figure is available in the directory archive FigM.tgz within the data archive available from https://ir.library.msstate.edu/bitstream/handle/11668/19731/Tice_etal.PhyloFisher.DATA.tgz.

**Fig N** Ratio of fast to slow taxa site rates, on a per site basis, estimated from our gt80 dataset with our output tree from this supermatrix inferred in IQ-TREE under LG+G4+F+C60+PMSF with an LG+G4+F+C20 input tree for generation of the PMSF site frequencies. Fast/slow taxa site ratios were estimated using the *heterotachy.py* utility. The null-distribution as estimated from the LG+G4+C60+F simulation (Fig M) was used to calculate p-values from the top 3000, 6000, and 9000 fast/slow ratios. Data associated with this figure is available in the directory archive FigN.tgz within the data archive available from https://ir.library.msstate.edu/bitstream/handle/11668/19731/Tice_etal.PhyloFisher.DATA.tgz.

**Fast Site removal from the 3000 (step0) and 6000 (step1) most heterotachious sites removed datasets**
As described in the *heterotachy.py* description above, 3000 sites were removed in a stepwise fashion from the whole (gt80trimal – 72632 site) dataset. With a p-value of 0.001 as calculated the step0 dataset has 3000 sites removed. With a p-value of 0.003 as calculated above, the step1 dataset has 6000 sites removed. From each of these datasets (Step0.3000.HeterotacheousSitesRemoved.phy and Step1.6000.HeterotacheousSitesRemoved.phy), the fastest evolving sites were removed in a 3000 site stepwise fashion as described above in the fast site removal description (“*fast_site_remover.py -m Step0.3000.HeterotacheousSitesRemoved.phy -tr public.gt80trimal.PMSF-c20inputtree.treefile -c 3000” and “fast_site_remover.py -m Step1.6000.HeterotacheousSitesRemoved.phy -tr public.gt80trimal.PMSF-c20inputtree.treefile -c 3000*”). From this, we arbitrarily chose the 6000 fast sites removed dataset to further analyze from both of the 3000 and 6000 heterotachy datasets. Phylogenetic trees were inferred from both of these datasets (Fig O).


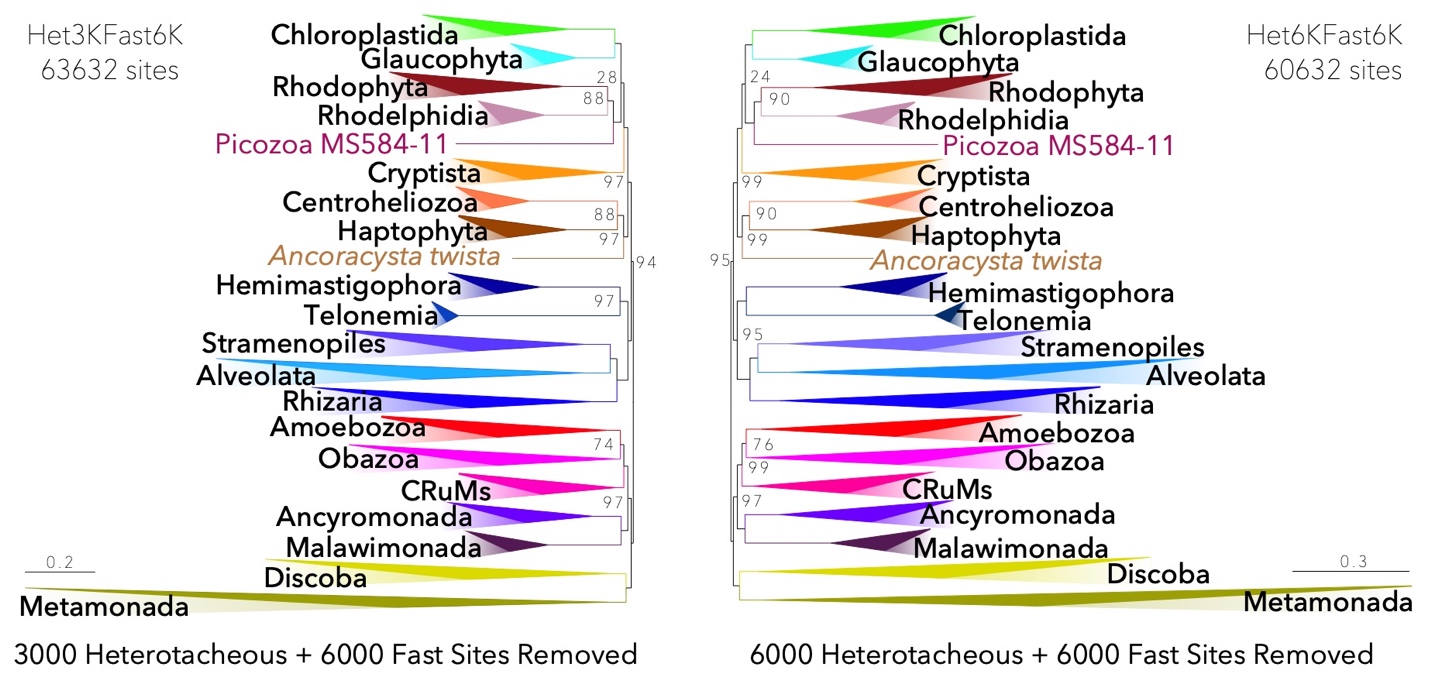


**Fig O** Heterotachious site removal of 3000 and 6000 sites from the whole dataset (72632 amino acid), removal of 3000 (p-value = 0.0001) (left) and 6000 (p-value = 0.003) (right). From these starting heterotachious removed datasets, 6000 of the fastest sites were removed using *fast_site_remover.py*, Het3KFast6K (63,632 sites) and Het6KFast6K (60,632 sites). ML tree was inferred for each dataset in IQ-TREE under LG+G4+F+C60+PMSF with an LG+G4+F+C20 input tree for generation of the PMSF site frequencies inferred in IQ-TREE with 1000 ultrafast bootstrap replicates (UFBOOT). UFBOOT values of 100% are not shown, all other values are indicated at their respective node. Data associated with this figure is available in the directory archive FigO.tgz within the data archive available from https://ir.library.msstate.edu/bitstream/handle/11668/19731/Tice_etal.PhyloFisher.DATA.tgz.

**Coalescent-based species tree using *astral_runner.py***

Coalescent-based species tree from the 240 ortholog trees inferred in RAxML (under the PROTCATLGF model with 100 bootstraps) were constructed using the default trimming methodology listed above in the *matrix_constructor.py* description. Tree was inferred by ASTRAL-III using the PhyloFisher utility, *astral_runner.py* (Fig P).


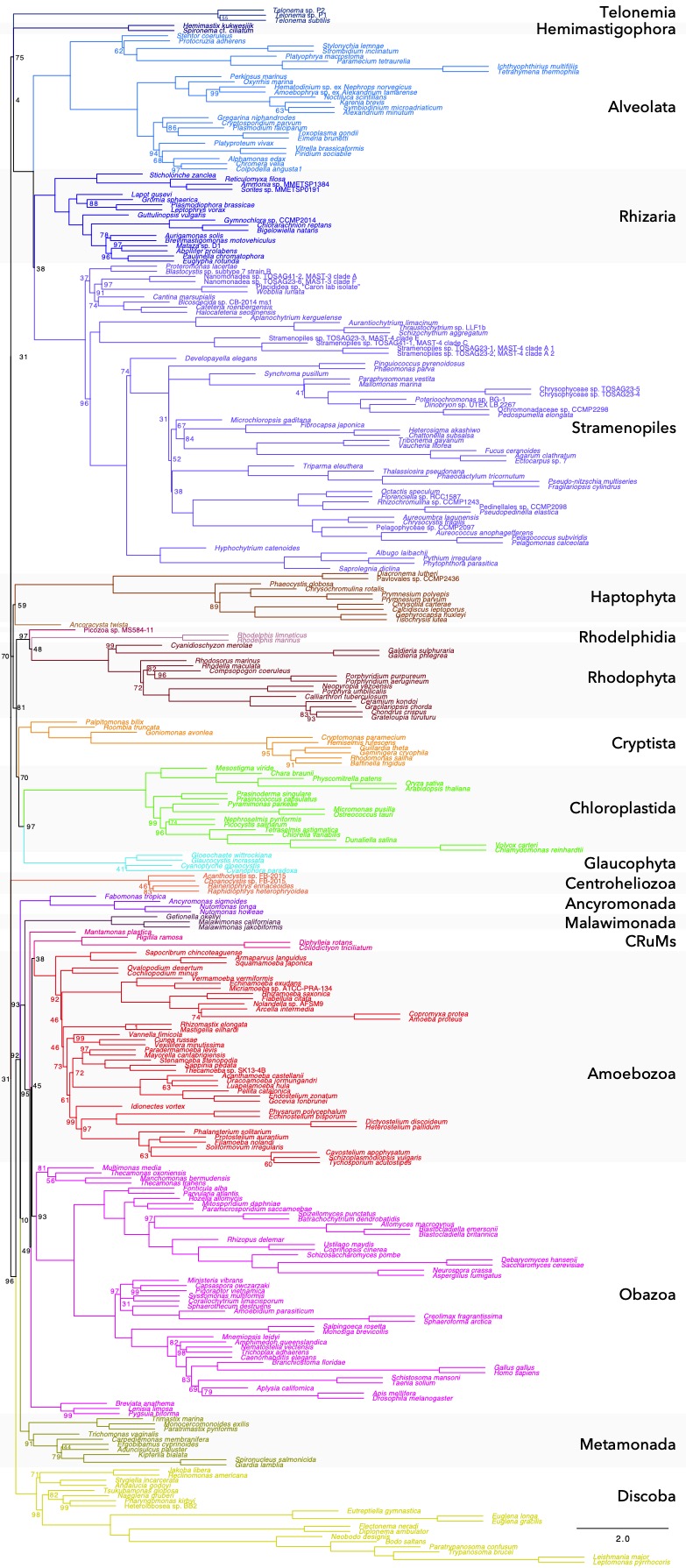


**Fig P** Coalescent-based species tree from the 240 ortholog trees inferred in RAxML (under the PROTCATLGF model with 100 bootstraps) using the default trimming methodology listed above in the *matrix_constructor.py* description. Tree was inferred by ASTRAL-III using the PhyloFisher utility, *astral_runner.py*. Values at nodes are ASTRAL bootstrap replicate values (BS). BS values of 100% are not shown, all other values are indicated at their respective node. Data associated with this figure is available in the directory archive FigP.tgz within the data archive available from https://ir.library.msstate.edu/bitstream/handle/11668/19731/Tice_etal.PhyloFisher.DATA.tgz.

**Binning orthologs based on their relative tree certainty (RTC) scores.** Trees were first inferred from the trimmed orthologs that resulted from *matrix_constructor.py*, {gene}.gt80trimal, in RAxML, using the command “raxmlHPC-PTHREADS-AVX2 -f a -T 2 -m PROTCATLGF -p 123 -x 123 -N 100 -s {gene}.gt80trimal.phy -n {gene}.gt80trimal” with 100 rapid bootstrap replicates. These were used as an input for RAxML to infer the RTC scores, using the command “raxmlHPC-PTHREADS-AVX2 -t RAxML_bipartitions.{gene}.gt80trimal -z RAxML_bootstrap.{gene}.gt80trimal -n IC.{gene}.gt80trimal -f i -m PROTCATLGF -T 2 -s NA”. The PhyloFisher package includes utility (*rtc_binner.py*) to generate RTC values and sort orthologs based on their RTC values. The utility collects top 25%, top 50%, top 75% and generates a supermatrix of each of these. Only the top 75% (180 ortholog) dataset was further examined. IQ-TREE under LG+G4+F+C60+PMSF with an LG+G4+F+C20 input tree for generation of the PMSF site frequencies inferred in IQ-TREE with 1000 ultrafast bootstrap replicates (MLBS) (Fig Q).

**
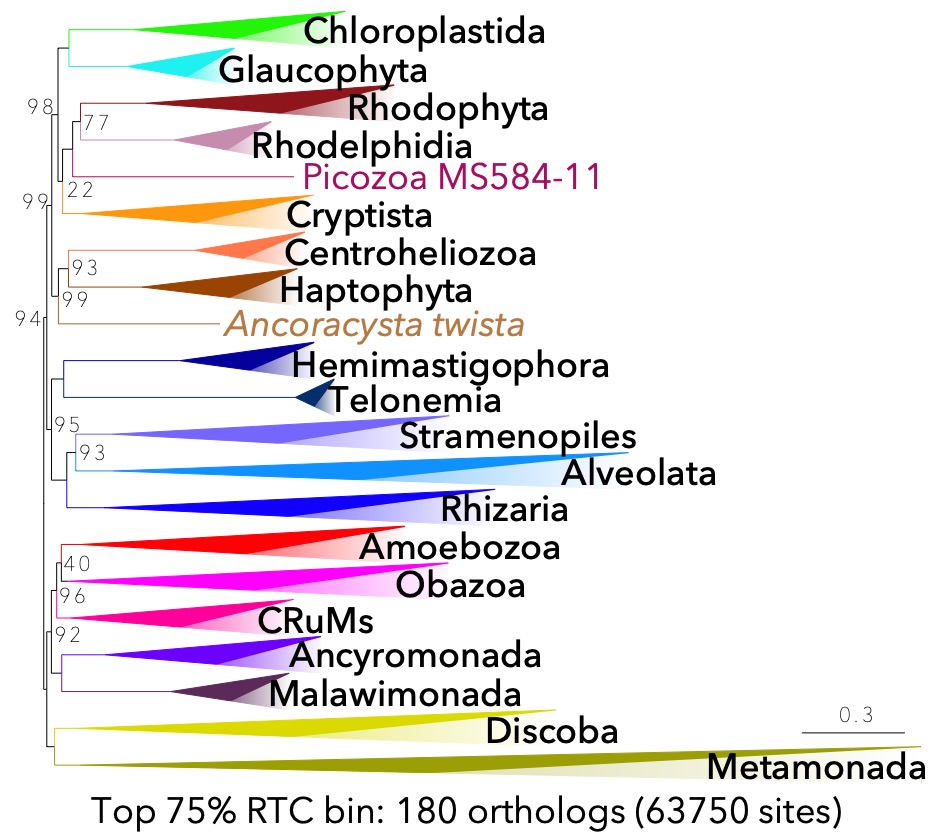
**

**Fig Q** Cartoon tree of the tree generated by RTC sorted bins (top 75%, 180 orthologs, 63750 sites) using *rtc_binner.py*. Input datasets for the *matrix_constructor.py* concatenation was gt80trimal single ortholog files. This tree was inferred in IQ-TREE under LG+G4+F+C60+PMSF with an LG+G4+F+C20 input tree for generation of the PMSF site frequencies inferred in IQ-TREE with 1000 ultrafast bootstrap replicates (UFBOOT). UFBOOT values of 100% are not shown, all other values are indicated at their respective node. Data associated with this figure is available in the directory archive FigQ.tgz within the data archive available from https://ir.library.msstate.edu/bitstream/handle/11668/19731/Tice_etal.PhyloFisher.DATA.tgz.

**Site heterogeneous modeling of the PhyloFisher dataset using mammal_modeler.py**

Proteins are known to have different evolutionary structural and functional constraints. This leads to the specific amino acid preferences at individual sites that may differ across the protein and a fixed homogeneous evolutionary model may lead to artifactual inferences. To combat this, site-heterogeneous models (e.g., CAT or C-series) are often used, but when using maximum likelihood approaches computational constraints typically mean that ML analyses will use fixed component frequency vectors inferred from external models (i.e., LG+G4+C60, etc.). Here we use MAMMaL v1.1.1 to estimate component frequency vectors directly from our phylogenomic matrix as described in [4] Because MAMMaL has constraints on the dataset with respect to naming and alignment type, we include a utility (*mammal_modeler.py*) that formats input data and calculates site-heterogeneous model derived from the users input supermatrix and an input tree from that supermatrix (“*mammal_modeler.py* -s public.gt80trimal.out -t public.gt80trimal.PMSF-c20inputtree.treefile -c 60 -at phylip”), where “-s” is supermatrix file, “-t” is the tree estimated from the supermatrix file, “-c” is the number of rate classes for MAMMaL to infer, and “-at” is the input alignment type. This tool runs MAMMaL without using likelihood weighting (“mammal -s seqfile -t treefile -c 60 -l”). The tool outputs site heterogeneous model evolution as a file called “esmodel.nex” in nexus format. This model was then used to estimate a tree in IQ-tree (“iqtree -nt 28 -m LG+ESmodel+G -mdef esmodel.nex -s public.gt80trimal.out -pre public.gt80trimal.LGMAMMaLC60GF -mem 160G”) and then this tree was used as an input tree for generation of PMSF site frequencies inferred in IQ-TREE with 1000 ultrafast bootstrap replicates (MLBS) (“iqtree -nt 28 -wbtl -bb 1000 -ft public.gt80trimal.LGMAMMaLC60GF.treefile -m LG+ESmodel+G -mdef esmodel.nex -s public.gt80trimal.out -pre public.gt80trimal.LGMAMMaLC60GF-LGMAMMaLC60GFPMSF -mem 800G”). Results from this analysis are shown in Fig R.


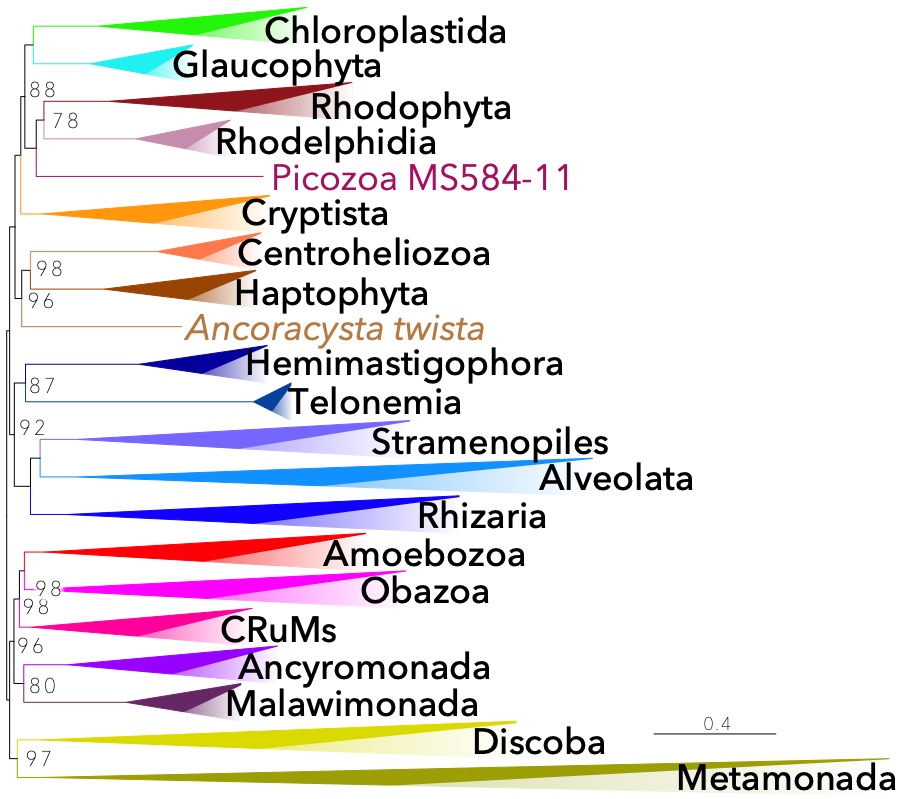


**Fig R** Cartoon tree of the tree generated in IQ-TREE using the model generated by *mammal_modeler.py*. This tree was inferred in IQ-TREE under LG+G4+F+ESmodel+PMSF (ESmodel = 60 rate classes estimated from the data inferred in MAMMaL) with an LG+G4+F+ESmodel input tree for generation of the PMSF site frequencies inferred in IQ-TREE with 1000 ultrafast bootstrap replicates (UFBOOT). Note, this is without -bnni UFBOOT correction due to a bug in IQ-TREE v1.6.12. UFBOOT values of 100% are not shown, all other values are indicated at their respective node. Data associated with this figure is available in the directory archive FigR.tgz within the data archive available from https://ir.library.msstate.edu/bitstream/handle/11668/19731/Tice_etal.PhyloFisher.DATA.tgz.

**Output single gene trees from BUSCO128 dataset [5] with inadvertent paralog inclusion.**
Single gene trees were inferred using the methods outlined in the main text for 128 of the top RTC scoring trees from the dataset of [5], using the *rtc_binner.py* script. In these 128 alignments (BUSCO128) we included the taxa and sequences from the dataset of [5] along with sequences that were collected via the fisher.py algorithm. Trees were processed by eye using *parasorter* and in 6 of the 128 genes paralogs were identified as included in the dataset of [5]. These tree outputs as viewed in *parasorter* are provided as Figs S-X.

**
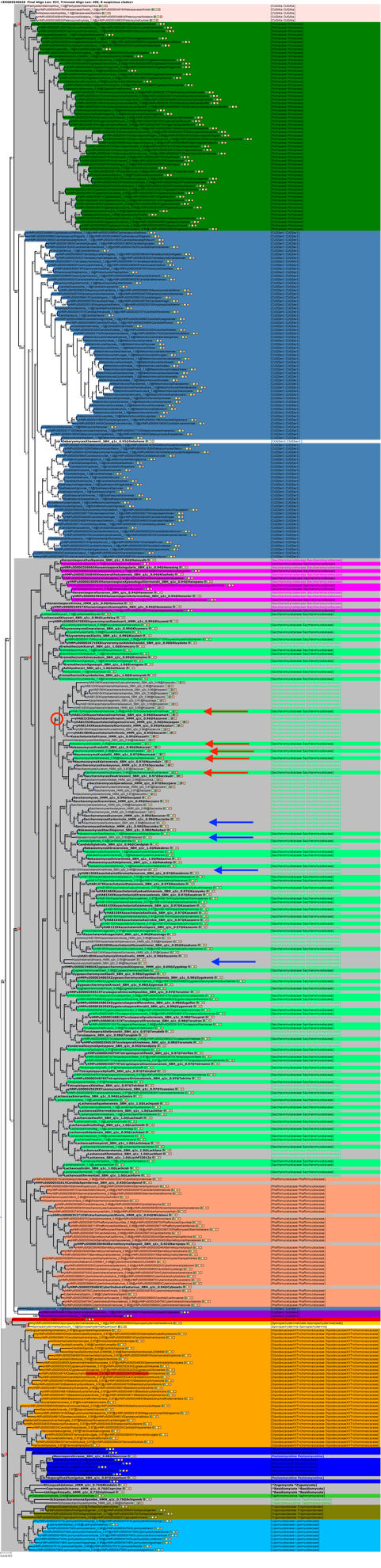
_­­­­_**

**Fig S** Phylogenetic tree of ortholog EOG0934062S of the dataset from [5]. Tree was inferred using the *sgt_contructor.py* as detailed in the main text. Tree is the output from *parasorter*. Leaf names in bold are identified by the fisher.py algorithm as suggested orthologs. Leaf names not bolded are from the sequences collected as potential paralogs. The leaves with a colored background are those sequences from the dataset from [5]. Problematic paralogs are highlighted with red arrows and the corrected replacement identified by PhyloFisher are highlighted by blue arrows. A downloadable figure and data associated with this figure is available in the directory archive FigS-X.tgz within the data archive available from https://ir.library.msstate.edu/bitstream/handle/11668/19731/Tice_etal.PhyloFisher.DATA.tgz.

**
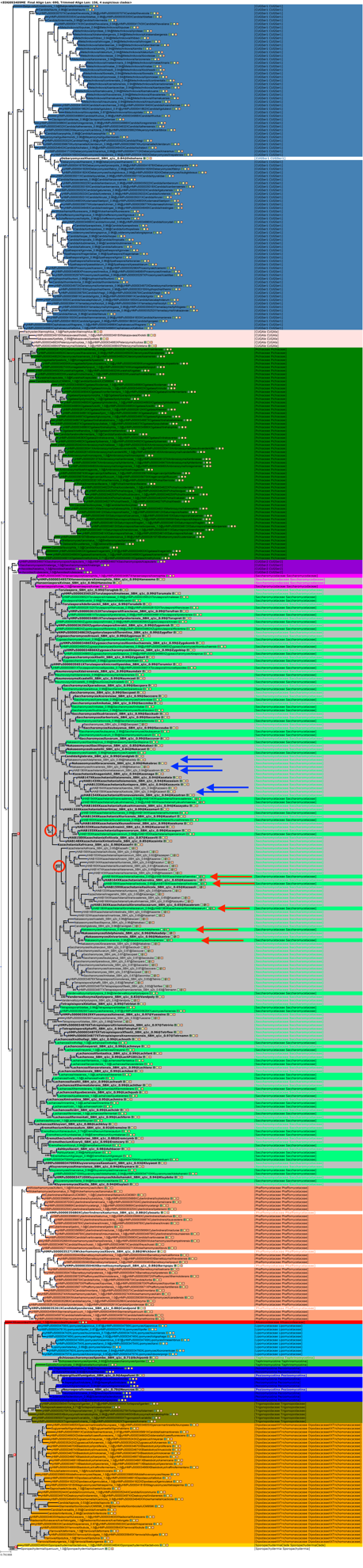
**

**Fig T** Phylogenetic tree of ortholog EOG093409ME of the dataset from [5]. Tree was inferred using the *sgt_contructor.py* as detailed in the main text. Tree is the output from *parasorter*. Leaf names in bold are identified by the fisher.py algorithm as suggested orthologs. Leaf names not bolded are from the sequences collected as potential paralogs. The leaves with a colored background are those sequences from the dataset from [5]. Problematic paralogs are highlighted with red arrows and the corrected replacement identified by PhyloFisher are highlighted by blue arrows. A downloadable figure and data associated with this figure is available in the directory archive FigS-X.tgz within the data archive available from https://ir.library.msstate.edu/bitstream/handle/11668/19731/Tice_etal.PhyloFisher.DATA.tgz.


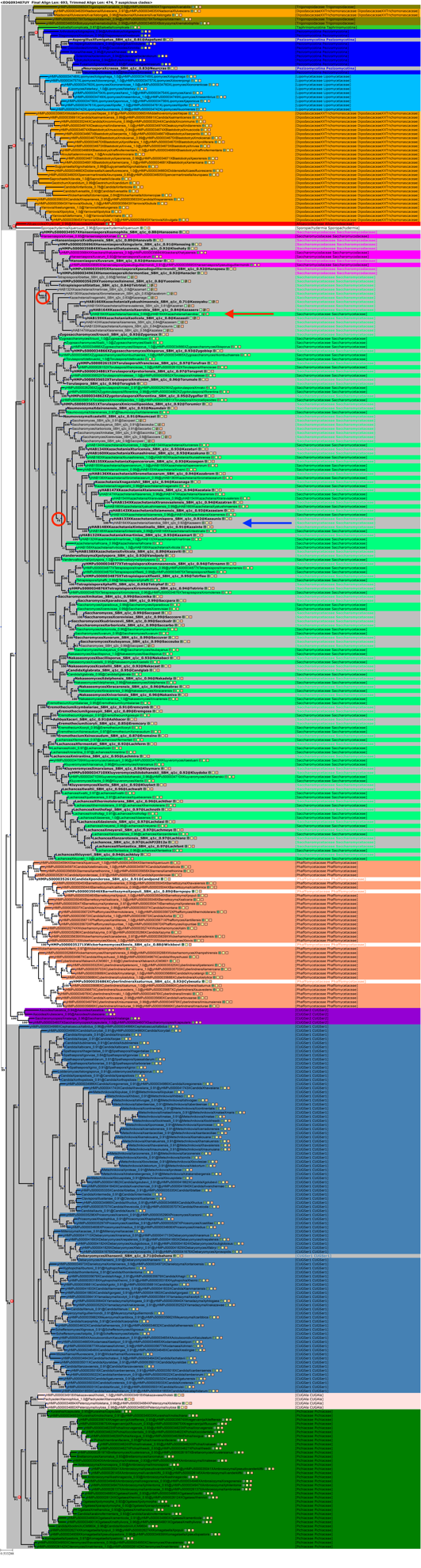


**Fig U** Phylogenetic tree of ortholog EOG093407UY of the dataset from [5]. Tree was inferred using the *sgt_contructor.py* as detailed in the main text. Tree is the output from *parasorter*. Leaf names in bold are identified by the fisher.py algorithm as suggested orthologs. Leaf names not bolded are from the sequences collected as potential paralogs. The leaves with a colored background are those sequences from the dataset from [5]. Problematic paralogs are highlighted with red arrows and the corrected replacement identified by PhyloFisher are highlighted by blue arrows. A downloadable figure and data associated with this figure is available in the directory archive FigS-X.tgz within the data archive available from https://ir.library.msstate.edu/bitstream/handle/11668/19731/Tice_etal.PhyloFisher.DATA.tgz.

**
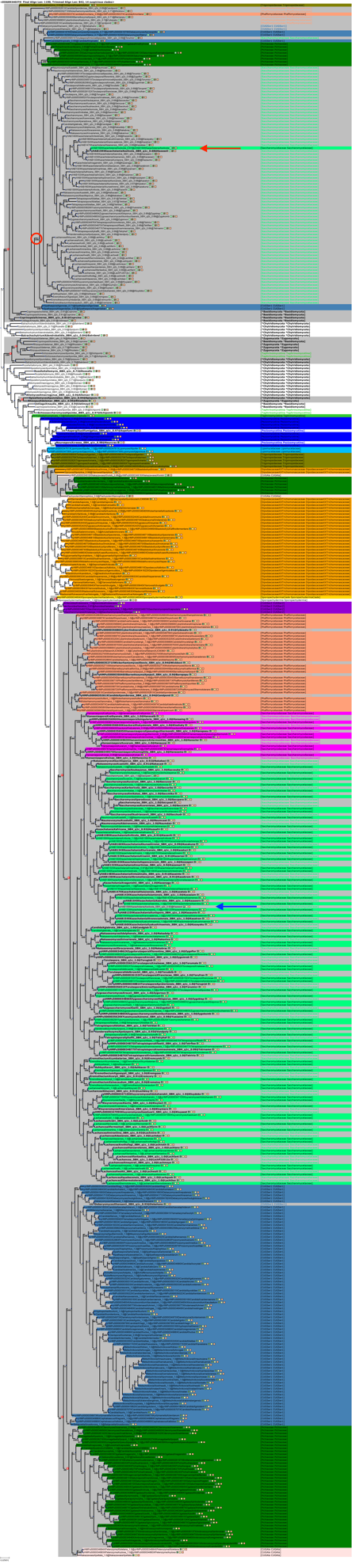
**

**Fig V** Phylogenetic tree of ortholog EOG093403TH of the dataset from [5]. Tree was inferred using the *sgt_contructor.py* as detailed in the main text. Tree is the output from *parasorter*. Leaf names in bold are identified by the fisher.py algorithm as suggested orthologs. Leaf names not bolded are from the sequences collected as potential paralogs. The leaves with a colored background are those sequences from the dataset from [5]. Problematic paralogs are highlighted with red arrows and the corrected replacement identified by PhyloFisher are highlighted by blue arrows. A downloadable figure and data associated with this figure is available in the directory archive FigS-X.tgz within the data archive available from https://ir.library.msstate.edu/bitstream/handle/11668/19731/Tice_etal.PhyloFisher.DATA.tgz.

**
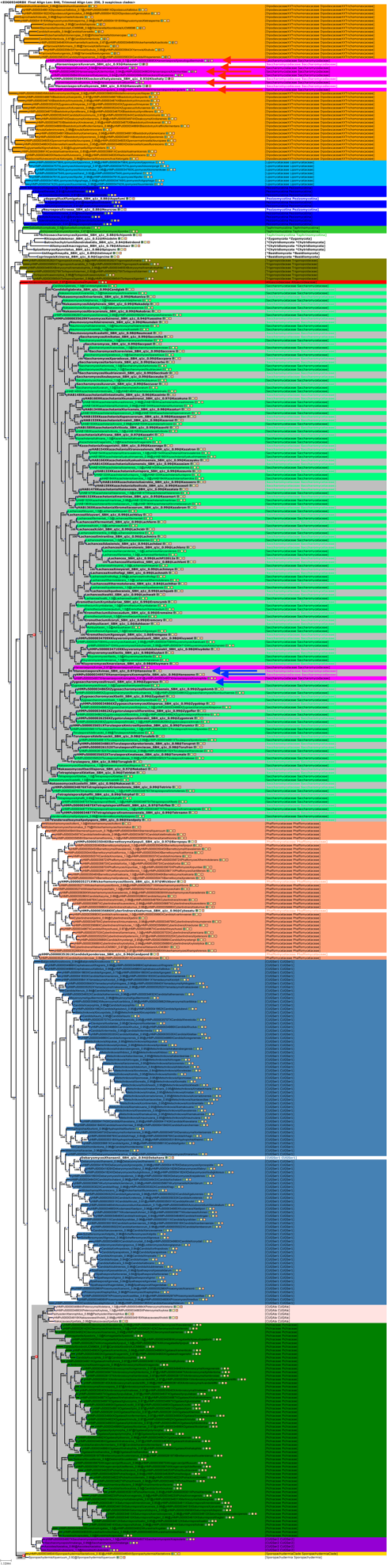
**

**Fig W** Phylogenetic tree of ortholog EOG09340RBX of the dataset from [5]. Tree was inferred using the *sgt_contructor.py* as detailed in the main text. Tree is the output from *parasorter*. Leaf names in bold are identified by the fisher.py algorithm as suggested orthologs. Leaf names not bolded are from the sequences collected as potential paralogs. The leaves with a colored background are those sequences from the dataset from [5]. Problematic paralogs are highlighted with red arrow. A downloadable figure and data associated with this figure is available in the directory archive FigS-X.tgz within the data archive available from https://ir.library.msstate.edu/bitstream/handle/11668/19731/Tice_etal.PhyloFisher.DATA.tgz.

**
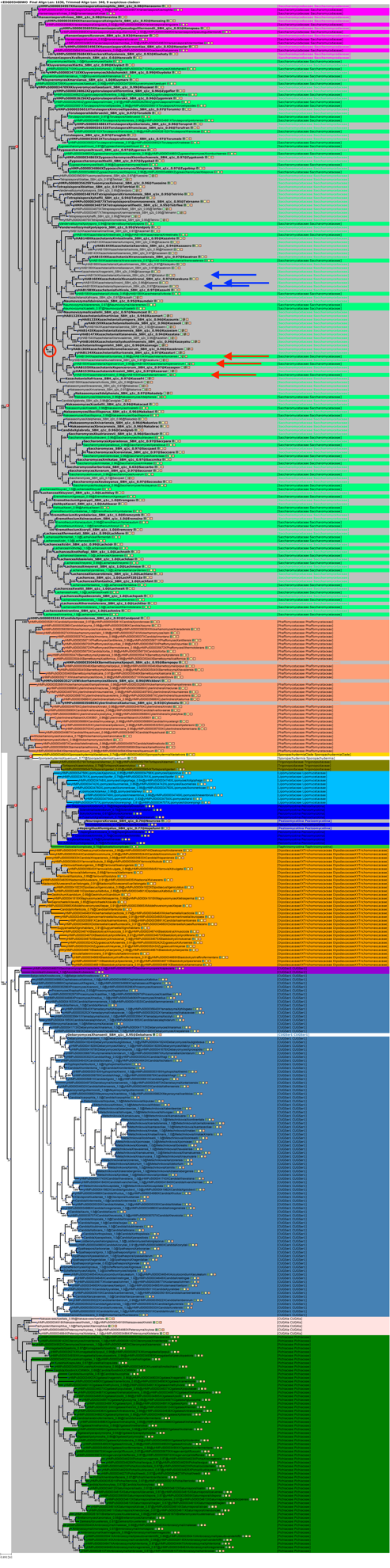
**

**Fig X** Phylogenetic tree of ortholog EOG093400WO of the dataset from [5]. Tree was inferred using the *sgt_contructor.py* as detailed in the main text. Tree is the output from *parasorter*. Leaf names in bold are identified by the fisher.py algorithm as suggested orthologs. Leaf names not bolded are from the sequences collected as potential paralogs. The leaves with a colored background are those sequences from the dataset from [5]. Problematic paralogs are highlighted with red arrows and the corrected replacement identified by PhyloFisher are highlighted by blue arrows. A downloadable figure and data associated with this figure is available in the directory archive FigS-X.tgz within the data archive available from https://ir.library.msstate.edu/bitstream/handle/11668/19731/Tice_etal.PhyloFisher.DATA.tgz.

**Fig Y** Phylogenetic reconstruction of the tree of Saccharomycetaceae using the PhyloFisher 208 dataset. Maximum likelihood tree built using (LG+G4+F+C60-PMSF model, with an LG+G4+F+C20 ML tree as a PMSF guide input tree) in IQ-TREE v1.6.7.1[1]. Sub-clades that make up the Saccharomycetaceae are shown in dark blue, while the outgroup clades of the Saccharomycodaceae and the Phaffomycetaceae are shown in dark green and cyan. Nodes are maximally supported (100 MLBS) unless otherwise shown. Data associated with this figure is available in the directory archive FigY.tgz within the data archive available from https://ir.library.msstate.edu/bitstream/handle/11668/19731/Tice_etal.PhyloFisher.DATA.tgz.

**Supplemental References**

1. Minh BQ, Schmidt HA, Chernomor O, Schrempf D, Woodhams MD, von Haeseler A, et al. IQ-TREE 2: New Models and Efficient Methods for Phylogenetic Inference in the Genomic Era. Molecular Biology and Evolution. 2020;37: 1530–1534. doi:10.1093/molbev/msaa015

2. Brown MW, Heiss AA, Kamikawa R, Inagaki Y, Yabuki A, Tice AK, et al. Phylogenomics Places Orphan Protistan Lineages in a Novel Eukaryotic Super-Group. Genome Biology and Evolution. 2018;10: 427–433. doi:10.1093/gbe/evy014

3. Strassert JFH, Jamy M, Mylnikov AP, Tikhonenkov DV, Burki F. New Phylogenomic Analysis of the Enigmatic Phylum Telonemia Further Resolves the Eukaryote Tree of Life. Molecular Biology and Evolution. 2019;36: 757–765. doi:10.1093/molbev/msz012

4. Susko E, Lincker L, Roger AJ. Accelerated Estimation of Frequency Classes in Site-Heterogeneous Profile Mixture Models. Molecular Biology and Evolution. 2018;35: 1266–1283. doi:10.1093/molbev/msy026

5. Shen X-X, Opulente DA, Kominek J, Zhou X, Steenwyk JL, Buh KV, et al. Tempo and Mode of Genome Evolution in the Budding Yeast Subphylum. Cell. 2018;175: 1533-1545.e20. doi:10.1016/j.cell.2018.10.023
