# Supplementary material for: PhyloFisher: A phylogenomic package for resolving eukaryotic relationships
Source: PLoS Biol. 2021 Aug 6;19(8):e3001365. doi: 10.1371/journal.pbio.3001365 (PMC8345874; doi:10.1371/journal.pbio.3001365)
Supplement: Attachment — Submitted filename: Tice_etal.PhyloFisher.Response.to.Reviewers_v4.pdf [file pbio.3001365.s003.pdf]

Dear Matt,

Thank you very much for submitting your manuscript "PhyloFisher: A phylogenomic package for resolving deep eukaryotic relationships" for consideration as a Community Page at PLOS Biology. Your manuscript has been evaluated by the PLOS Biology editors, and by three independent reviewers.

IMPORTANT: Many thanks for your patience while we discussed the reviews. We took a little extra time as we became concerned about whether a Community Page is the right format for this. I'd wondered this when you first approached us before submission, and we went back and forth between Community Page and a Methods and Resources paper. Recently we have started to refocus our Community Pages (and other "magazine section" formats) to make them shorter and of broader appeal. It is also clear from the reviewers (and especially reviewers #1 and #2) feel that the paper needs to be expanded somewhat to attend to their concerns.

You'll see that reviewers #1 and #2 are pushing for the authors to demonstrate the pipeline being put through its paces on shallower trees, in order to broaden the appeal and utility of the method. Each of these reviewers had some additional, more minor concerns. Most of reviewer #3's requests relate to their inability to test the pipeline and the paucity of documentation on Github, rather than the paper per se.

We took the liberty of discussing the rationale of changing the paper to a Methods paper with an Academic Editor with relevant expertise, and they were supportive. We therefore ask that you convert your article (on re-submission) to a Methods and Resources paper (do check the article processing charges: https://secure-
web.cisco.com/1eaULkIQr9vIds9lvbtNocbwf9XBHvZhFiP6NaO6cBv2Y2ScxEP1V3a6fpyRXe
bZjNHsRbSxXuQL-
pyyoavLaWTXpkzvhuN9LUR1v1ux6ORpXj1lktkifYO5TdzkYC2qtsUZmikhckqR9RjZJMLk7
MPSZNjkF1wItyAjmfyDSJ_84ePGWRF57jYBxbf4N082mkeNl6BWsbueidkiBwKpFBWclBjlc
8z1_PRxtxb7NK2xl74u4ZcPkWg_k3TEFM4I14zmrWnov-
oFSJ9zTLLg_6b0MStjYLr4xfSfZqh1gnVinXHhoJ7X3TfpgO4Qax8GPCacg0Lcx2PPCkjCWVt
ZEKg/https%3A%2F%2Fplos.org%2Fpublish%2Ffees%2F); this would allow you to expand your proof-of-principle to address the reviewers' concerns, and you would also have the option of shunting some of the rather large Supplementary Info into the main article, giving it a more conventional structure.

In light of the reviews (below), we will not be able to accept the current version of the manuscript, but we would welcome re-submission of a much-revised version that takes into account the reviewers' comments. We cannot make any decision about publication until we have seen the revised manuscript and your response to the reviewers' comments. Your revised manuscript is also likely to be sent for further evaluation by the reviewers.

We expect to receive your revised manuscript within 3 months.

Matthew W. Brown, Ph.D. • 662.325.2406 • mwb250@msstate.edu • 007 Harned Hall
http://amoeba.msstate.edu

Please email us (plosbiology@plos.org<mailto:plosbiology@plos.org>) if you have any questions or coextension. At this stage, your manuscript remains formally under active consideration at our journal; please notify us by email if you do not intend to submit a revision so that we may end consideration of the manuscript at PLOS Biology.

IMPORTANT - SUBMITTING YOUR REVISION

Your revisions should address the specific points made by each reviewer. Please submit the following files along with your revised manuscript:

1. A 'Response to Reviewers' file - this should detail your responses to the editorial requests, present a point-by-point response to all of the reviewers' comments, and indicate the changes made to the manuscript. *NOTE: In your point by point response to the reviewers, please provide the full context of each review. Do not selectively quote paragraphs or sentences to reply to. The entire set of reviewer comments should be present in full and each specific point should be responded to individually, point by point. You should also cite any additional relevant literature that has been published since the original submission and mention any additional citations in your response.

2. In addition to a clean copy of the manuscript, please also upload a 'track-changes' version of your manuscript that specifies the edits made. This should be uploaded as a "Related" file type.

Re-submission Checklist When you are ready to resubmit your revised manuscript, please refer to this re-submission checklist: https://secure-web.cisco.com/1BI9A1x_xQAECjBywkadqASjzPIVT0lw8cRrubrljftx7gpszq03E-7DZ6IyLDeeVX1jjPJBBlNNq4CojgRl0GOeqq_CU5cSv30dXq3hOFqlv_7NUaZ6nFcZBQZYS_cho2L319t9mvLpw33a0W5ZSfp2h4u3t3fmpKzlkmnfAp6HMJxCM_iACGYW6IAivaXLlYDvEecpgksnTNGOq2sAOXGkkJ1kvOAdSqKfhk7FQZmu0XCnOhPDMAfpsikXjshfDWFOGhCxASpGyLgiZjtJZigNb0CV72Z8RMv12QFo-lpzMduJgwDG7p9hqUYsgSVYy/https%3A%2F%2Fplos.io%2FBiology_Checklist

To submit a revised version of your manuscript, please go to https://secure-web.cisco.com/1-ZtF9hzuzDnN2V1cXLUzKpOFbpcIvwRkaFUq3Sb2BJ0q9L7PBSxpNiT0dLQqZvoQx3M-o6RDFysUL6WYYMFDvFILDFzEWuqrBvZfzV24_piz7P0k-hkzaHIuUN-z6siP9PROpR2kROguvhcavpBORyR8sN8Mpbw2Vt11nv_LYoLYR3f-le8ASPQZJTruXolDUhlgKPAaKFNV9X6e6N7J3YH8_oYNIHr_hy4CU-jRbFSYu96caEv-C6bDvqiARjJUjiFvlfXWmKdodZhoTINPZ13E9hnRq-u3VZu2BZX7-308iIvG0dru_L_Riri5gR4UzLFKKmc3cg8WW-3UIpEh_g/https%3A%2F%2Fwww.editorialmanager.com%2Fpbiology%2F and log in as an Author. Click the link labelled 'Submissions Needing Revision' where you will find your submission record.

Please make sure to read the following important policies and guidelines while preparing your revision:

Published Peer Review Please note while forming your response, if your article is accepted, you may have the opportunity to make the peer review history publicly available. The record will

include editor decision letters (with reviews) and your responses to reviewer comments. If eligible, we will contact you to opt in or out. Please see here for more details: https://secure-web.cisco.com/1bbM5IuqvpGW2_OV7sTflOKCTV96q9X_WmJibQM44un7D1dXMQ_F1bxTRJJ8U0Js4viwTStLbDCfvdxaeV9JQE-Q9AiH9Tfb9iSa2tfJzb4kfXQbEIhnUHNMXRiNpt3TJFz432m4sZWVHOhIoMqrV5-2C01RQ2Ca8lWd74NW_-ZN9WFQfFHlAsjfAxny4wYDX68kd-iESXtL_2dCDxKZ6R1BCAwVceRsXDyDHo0ZWlPYZbfwyjrqcjXnJRBRPSenU4ULOw0AGTmhvLttYUignJRGFs-IkczgxWbVpdm8lSa7MVPcYMJDUHO3GxtkmYHv6s8OmCiMiBERCbgQ_LqP0Ag/https%3A%2F%2Fblogs.plos.org%2Fplos%2F2019%2F05%2Fplos-journals-now-open-for-published-peer-review%2F

PLOS Data Policy Please note that as a condition of publication PLOS' data policy (http://secure-web.cisco.com/1XB0Ca9_1WqJ1NCHUsewraqL8mKRIw_mt7KkrLz6rl4CEEpemYYwMI-ZtOa0uSGd8aYZWmgGLgA9zFd-dEeu8XacZV1R1auzGpOpYXisaj6ic_oTSOgufYb-4vF0qxswULbZoCBQWiQ5EMAcM12SFPLgKyRe5O2fX1d-AdNSE2Q893BHyzVYwKKxmp_ssrKYEhfAidzJfD0Vvez3diO6ACw-Hbv3NP3H7czHejjGbIvwxRLwy4HXs0-iqg3gRSih4NaeHGwua_IuE0VskRhiL6itlzxkogUXBbHnkB85YL3OFcSzOXBu5cFmvFoAcWMlIwiOpOgdX6Tq9rU8qDwlwKA/http%3A%2F%2Fjournals.plos.org%2Fplosbiology%2Fs%2Fdata-availability) requires that you make available all data used to draw the conclusions arrived at in your manuscript. If you have not already dinclude any data used in your manuscript either in appropriate repositories, within the body of the manuscript, or as supporting information (N.B. this includes any numerical values that were used to generate graphs, histograms etc.). For an example see here: http://secure-web.cisco.com/1SMUd-k-LEQ1zOIQHqe44i_Toxnf0fKmLyJ17JXIAdi_hJzozVDdV-9vGpz4CrsxoL3r_Ujcxt7315IkPEE5eKtWXCxT_ZHWoFyngG4gXzDM25CSXTEDnhv2Vc5Qa5pW5a6l2fCj_4I3-vVkcLlpt7LuOopjuZRDeCQNGLKyqBHhC_v-9K4fYA60kVW20TGyI89wuVRMAbgt7SHfxlIK4f5wwRV4_JVSKgpuxJG31U5te8LZH6RM4ZAnNBTAn0P2VCzy1qfE19DQhZAdpnfwhm7ku_vlt1heDzJ6nH5XBXF_PIyynOD4g5m2u4oVTG-tk/http%3A%2F%2Fwww.plosbiology.org%2Farticle%2Finfo%253Adoi%252F10.1371%252Fjournal.pbio.1001908%23s5

Blot and Gel Data Policy We require the original, uncropped and minimally adjusted images supporting all blot and gel results reported in an article's figures or Supporting Information files. We will require these files before a manuscript can be accepted so please prepare them now, if you have not already uploaded them. Please carefully read our guidelines for how to prepare and upload this data: https://secure-web.cisco.com/1PPZcaiU3LV4c7B8-DC2Y4dMlwcyjj1-YbhdM2AtgHNEGHWvtJcaCY31J10mqKXDzl-HzqTIHs6kwIQkuvSNhKvgwKNvLX_8A9mkcCMNzF9f7Lpn_k3IPXbhsDpRxm9sm3bDnJOxtgakBxYv9mZBrdISBPG8fnWisR5nuBImWejO_zfINs6WFAKfbLdHIVSBbYcpRngg3z4ATv5Rul6RDeOR_hvpIaWMQS39s4koh-KDBgtpCLj5hpkFoRjSyWvekKGr9E5FiB18HYHeGX7T_pVTMgxfezk5_rnQSXXCxNwuxOlab-

m6mQkqbLdrqxak5oxnqROqZifFwg1U_34J2Tw/https%3A%2F%2Fjournals.plos.org%2Fplosbiology%2Fs%2Ffigures%23loc-blot-and-gel-reporting-requirements

Protocols deposition To enhance the reproducibility of your results, we recommend that if applicable you deposit your laboratory protocols in protocols.io, where a protocol can be assigned its own identifier (DOI) such that it can be cited independently in the future. For instructions see: https://secure-web.cisco.com/1RuJZfkxsRnDECq3SkkAUDb_88bIu4WDMefPFOF8E6gDngTAiveH_qFejXeVNAeN7FtyfIF8laHOTjSQtHKw2pFbRWaG31M-xUPAFTDcQBtSlHO3wWBn5hsn1ICZNaLvEoDI2ngdmRPlYe-hdaVEzDuy52g_4Ha9mV4PVW3bqD-T6gm50IiyiXqNkSkdna_Ae5KlJ9fxG99Z9nm1qNelWchvMVDs3YcWZsV0mIkSERHHIbj78jsE0Xb5yV0DwuIheydiMJY2lnIjd8tL9rNHxogUimipPLdVbSJ2RGpdyFJQ8lTf4X6aA8RKbfa-h_pO68utWPJNHE8X6UW3w_k8T6g/https%3A%2F%2Fjournals.plos.org%2Fplosbiology%2Fs%2Fsubmission-guidelines%23loc-materials-and-methods

Thank you again for your submission to our journal. We hope that our editorial process has been constructive thus far, and we welcome your feedback at any time. Please don't hesitate to contact us if you have any questions or comments.

Sincerely,

Roli

Roland G Roberts, PhD, Senior Editor, rroberts@plos.org<mailto:rroberts@plos.org>, PLOS Biology

****
REVIEWERS' COMMENTS:

IMPORTANT:

Please change your article type to "Methods and Resources" when re-submitting.

We have changed the article type as requested.

Reviewer #1:

The manuscript entitled "PhyloFisher: A phylogenomic package for resolving deep eukaryotic relationships" presents the software PhyloFisher that aims to facilitate phylogenomics analyses using the state of the art implementations. Such software in general comes in handy, espacially for those who are new in the field and have not their main research focus on resolving phylogenies. The software is scalable and different post-phylogeny analyses are implemented. It is helpful to combine the knowledge gained in the past years of phylogenomic, large scale analyses and make

them available to a broader user spectrum. I get the feeling that this software can also be used well in teaching, which is not mentioned in the manuscript. Overall the manuscript is very well written and understandable for the broader community the software is aimed for to be used. Therfore, I support the publication of the manuscript after some changes and add-ons have been performed.

Thank you for your kind words and critical suggestions on how we can improve our manuscript. We have added a paragraph in the discussion section promoting the use of PhyloFisher as a training tool for teaching phylogenomics. This is a wonderful point and not something we had previously considered.

My main advice is two apply PhyloFisher to a different area of the tree of life to show the full potential of the software. The single example provided is addressing a very deep node. I suggest to add an additional analysis for a much more internal node within plants, animals or such.

We have now added additional phylogenomic analyses reconstructing the Saccharomycetaceae clade of budding yeast. This particular group of yeasts has been diverging for ~100 million years and has a genetic diversity similar to what is seen in flowering plants. Our phylogenomic reconstructions using PhyloFisher's provided set of orthologs and two separate datasets that represent a subset of data used in Shen et al. 2018 that were assembled and processed with PhyloFisher are consistent with the original study of Shen et al. 2018. Additionally, we have requested that the authors Shen and Rokas be involved in our study and have included them here as co-authors. They aided our team in developing a strategy to address PhyloFisher's proof of concept in use shallow-level phylogenomics.

Shen XX, Opulente DA, Kominek J, Zhou X, Steenwyk JL, Buh KV, Haase MAB, Wisecaver JH, Wang M, Doering DT, Boudouris JT, Schneider RM, Langdon QK, Ohkuma M, Endoh R, Takashima M, Manabe RI, Čadež N, Libkind D, Rosa CA, DeVirgilio J, Hulfachor AB, Groenewald M, Kurtzman CP, Hittinger CT, Rokas A. Tempo and Mode of Genome Evolution in the Budding Yeast Subphylum. *Cell*. 2018 Nov 29;175(6):1533-1545.e20. doi: 10.1016/j.cell.2018.10.023. Epub 2018 Nov 8. PMID: 30415838; PMCID: PMC6291210.

Also, it would be good to show PhyloFishers performance on the gene selection, orthology determination of very new datasets of such internal branched. As it is known, there are many, lineage specific genes that are informative to resolve phylogenetic relationships that are not part of the curated set of genes the authors have implemented. I guess this should be possible within revision time.

We apologize if we have been unclear regarding the functionality of PhyloFisher. PhyloFisher does not take an input set of proteomes and select a unique ortholog set for use in phylogenomic dataset construction. Rather, users who do not wish to use the provided set of genes have the option of providing any number of individual ortholog files (with an option to provide known paralogs) as input and the PhyloFisher script *build_database.py*. The script will generate the data necessary for the retrieval of the ortholog set from new taxa and subsequent analyses within the PhyloFisher workflow. The only exceptions being if a provided ortholog is assigned "no group" or to an

exclusively bacterial group in OrthoMCL. In these cases, the particular ortholog cannot be used in the PhyloFisher workflow.

With regards to PhyloFisher's performance on gene selection, we hope we have demonstrated through our re-analysis of the yeast datasets the potential of PhyloFisher to reveal previously unknown paralogy emerging at shallow and deep points in evolutionary time that was not detected using widely utilized methods such as collection of BUSCOs or all vs. all BLAST searches followed by OrthoMCL clustering, and automated homolog tree pruning.

The main Figure 1 can be better organized. It is quite compressed, the use of fonts is not easy to grab and a better design would be more helpful.

We agree the Figure 1 is quite busy and cold be difficult to follow. In the revised version we have reduced the amount of text in the figure, changed lines to arrows in order to show directionality in the workflow steps, and shaded main workflow step boxes in grey. Additionally, we have spread out the various items in the figure.

Furthermore, the colors used are not all discriminable by color-blind readers, so please use different color sets. This BTW is true for ALL figures incl. the Supplementary Material.

We apologize for this oversight and we completely agree. While we have chosen not to change the color scheme of taxonomic groups in Figure 3 as they are labeled, and interpretation is not reliant on the colors. To address this oversight throughout, every figure main and supplemental texts now has labels so color alone is not the only distinguishing element for different aspects in the figures. Within PhyloFisher itself, users have the option to choose their own color sets for the graphical aspects of the pipeline to accommodate for the different types of color-blindness.

Reviewer #2:

Tice et al. describe a pipeline for building and curating large multi-gene phylogenetic datasets for inferring the phylogeny of eukaryotes. The pipeline and reference dataset are likely to be used by members of this community. The scripts are well described and feature-rich. It seems that the main purpose of the pipeline is for adding species to their database of 300 eukaryotes and 240 genes. They have used this dataset to successfully infer the tree of eukaryotes, with a focus on inferring deep splits in the tree. Researchers who wish to do this will find the approach described here very appealing. It appears that one could sequence the transcriptome or genome of a eukaryote and fairly easily place it in their tree of eukaryotes without having to master every step of performing a phylogenomic analysis, which for most people involves lots of scripting and mastering of dozens of software programs. Thus, this set of scripts appears to make this endeavor more accessible to the broader eukaryotic phylogenomics community.

It is less clear to me how useful this will be for someone who works on a particular group of eukaryotes. For example, the myBaits angiosperm 353 kit has found widespread use by the flowering plant community. Could they create a database of these orthologs and have a parallel "angiosperm" version of the pipeline? Is it flexible in this way, or would that be a lot of work? Could this easily be adopted for vertebrates? Green algae? Beetles?

We apologize for our lack of clarity when describing the functionality of PhyloFisher. It is correct that if a user wished to use the tools provided in PhyloFisher to construct a custom database using the myBaits angiosperm 353 kit gene set and then retrieve this ortholog set from additional taxa they could have a parallel angiosperm version of the pipeline. The only exception would be if any of the orthologs within the "myBaits angiosperm 353 kit" were assigned "no group" in BLAST searches against OrthoMCL v.5.0. These orthologs would not be usable in the PhyloFisher workflow. We detail the construction of such custom databases in the PhyloFisher manual we have now provided in our resubmission.

As also requested by reviewer 1, we show that PhyloFisher is a tool that can be used to perform phylogenomic analyses of a particular group of eukaryotes and is not limited to eukaryote wide phylogenomic analyses. To do this, we have added additional phylogenomic analyses reconstructing the Saccharomycetaceae clade of budding yeast. This particular group of yeasts has been diverging for ~100 million years and has a genetic diversity similar to what is seen in flowering plants. Our additional phylogenomic reconstructions using both PhyloFisher's provided set of orthologs and two separate datasets from a subset of data used in Shen et al. 2018 that were generated and processed with PhyloFisher are consistent with the aforementioned study that included this clade of budding yeasts.

This paper emphasizes manual curation of phylogenomic datasets, referring to manual intervention and inspection as "absolutely required" (line 64). I'm not sure I agree, but one advantage of their pipeline is that it reportedly logs the manual curation steps, which is important for reproducibility, which is a major problem in phylogenomics. Anyone who's carried out these types of analyses can recognize a Methods section where it was clear that the investigator made lots of undocumented decisions. I think this manuscript somewhat trivializes the tree-pruning algorithms of Yang and Smith (reference 6 in this manuscript). That pipeline does not have all of the features of this one, to be sure, but it semi-automates many of the same steps, and it follows fixed algorithms to identify and prune paralogs, giving orthologs for gene-tree and then species-tree inference. This was probably one of the biggest advances in the field for de novo phylogenomics. This approach differs in that it is not de novo, rather one might use the approach of Yang and Smith to obtain a set of orthologs de novo, create a database of these, then adopt parts of the pipeline described here to complete the analysis or for future studies. If I have mischaracterized this, then I apologize, but I'd encourage you to clarify the manuscript. This is not for de novo phylogenomics, correct?

It is correct to assume our approach is not for de novo phylogenomics. It is also correct to assume that users wanting to create their own custom database could use the steps of the Yang and Smith pipeline to acquire an initial ortholog set for use in the PhyloFisher workflow. We have added text to the introduction to clarify that PhyloFisher, at minimum, requires a predefined set of orthologs (with the option to provide known paralogs) to subsequently retrieve the gene set from new taxa.

In a related question, how independent are the various scripts in the pipeline, i.e., could one use just the scripts for setting up their Astral run or stripping high-rate sites, or would this throw a ton of errors because they haven't used the entire pipeline from step 1? I hope it's the former because some of these scripts will be very useful.

We have designed the utility scripts (such as the tools that remove fast sites and aid in Astral runs also others outlined in main text Table 1) provided in the package to act as "stand-alone" programs so we do not prevent their use by systematist who decide the main PhyloFisher workflow does not fit their needs but are using standard file types (fasta, phylip, nexus) and performing some or all of the analyses or dataset permutations our provided utility scripts facilitate. In fact, the utility scripts *fast_site_remover.py*, *random_gene_resampler.py*, and *heterotachy.py* were used to perform their associated functions on the phylogenomic dataset of Salomaki et al. 2021 that was created outside the PhyloFisher workflow.

However, the main workflow scripts are dependent on the user having processed their data with the preceding main workflow scripts. The one exception is our provided script for homolog tree construction (*sgt_constructor.py*). We have provided *sgt_constructor.py* as a means for less experienced users to filter, align, trim and construct phylogenetic trees in a rigorous fashion from homologs in a single simple step. However, we realize advanced users may want to use alternative strategies, parameters, programs, models of evolution, etc for these steps of a standard phylogenomic workflow. Knowing this we have made it easy to re-enter the main PhyloFisher workflow if users choose to circumvent this particular script.

Salomaki ED, Terpis KX, Rueckert S, Kotyk M, Varadínová ZK, Čepička I, Lane CE, Kolisko M. Gregarine single-cell transcriptomics reveals differential mitochondrial remodeling and adaptation in apicomplexans. *BMC Biol* 19, 77 (2021). https://doi.org/10.1186/s12915-021-01007-2

I mentioned above that I disagree somewhat that manual intervention is "absolutely required." I would also argue that (my understanding of) their description of a "phylogenetically informed/aware" approach to ortholog selection makes it sounds much more sophisticated than it actually is. "Phylogenetically informed" means that the user has identified a close relative in the reference dataset, so the HMM or BLAST searches are tailored accordingly.

It was certainly not our intent to inflate the sophistication of our ortholog collection algorithm. However, more steps are involved than simply using a particular related taxon's peptides as queries in the subsequent BLAST searches. The "phylogenetically informed/aware" route also uses FastTree in conjunction with taxonomy provided in the metadata.tsv and input_metadata.tsv files to prioritize and eliminate sequences that are collected in the initial HMM and BLAST searches of the algorithm. A detailed description of logic applied in the phylogenetically informed path through *fisher.py* is provided in the "phylogenetically informed route" section of the main text materials and methods as well as the "ortholog selection via the fisher algorithm" section of provided manual.

This is related to my friendly disagreement about manual curation as an "absolute" requirement (again, their language). In many of the papers on eukaryote phylogeny, some newly discovered or newly cultured taxon is sequenced, added to a tree, and there's a big story about it being sister to one lineage or another. These trees have dozens or hundreds of taxa, like the one in the present manuscript, so these are often deep splitting lone branches on the tree, the kind of branch that is often (and wrongfully) described as basal. So what does manual inspection of the gene trees do for you in this example? Sometimes an orthogroup includes deep-branching out-paralogs, and oftentimes single gene trees have abysmally low bootstrap (or other) support. When dealing with an organism whose phylogenetic placement is truly unknown and with hundreds of gene trees with low or variable support, is it really best practice to have the user manually selecting which sequences are orthologs and which are paralogs? I'd rather have an approach like Yang and Smith make those unbiased determinations for me. I'm not arguing here that Yang and Smith is perfect? But manual determination of paralogs for deep nodes in the eukaryotic phylogeny seems potentially fraught.

We agree these automated tree pruning algorithms could be very useful and dramatically reduce the amount of time required for data analysis. We also agree they could increase reproducibility, reduce human bias, eliminate the need for tree parsing to be done by experts in a particular group(s), and make it feasible to use much larger datasets for phylogenomic analyses due to lack of need for manual curation of every tree. However, we believe our reanalysis of the Shen et al. 2018 dataset demonstrates that these algorithms are not sophisticated enough to trust “blindly” without manual inspection at some point. While we cannot exclusively blame the automated tree pruning algorithm applied to the original dataset of Shen et al. for the inclusion of paralogs in the final dataset, our discovery of these accidental inclusions does allow us to say the algorithm did not prevent them.

We are also concerned that these algorithms do not appear to be able to account for contamination in input data. Thus, it remains unclear to us how much loss of correct data (due to perceived duplications) and/or retention of contaminating sequences occurs when these algorithms are applied without manual inspection occurring beforehand if contaminated data is accidentally used as input. Transcriptomes (especially those generated using single cell methods to collect data from organisms in environmental samples) are notoriously contaminated but often make up the majority of available “omic-level” data used in phylogenomic studies. Knowing that the inclusion of a small number of non-orthologous sequences in phylogenomic datasets can influence contentious branching patterns we prefer to examine this aspect of the data by eye and encourage others to do so as well.

Even with our above arguments in support of manual inspection of homolog trees we have toned down our previous language from “absolutely” to “highly recommended.”

This was a well-written manuscript by a group that has clearly thought about these issues and has the benefit of experience. I think the protist and eukaryote phylogeny communities are likely to adopt these pipelines for their work, making them potentially very impactful. If the answers to all of my outstanding questions turn out to have favorable answers, some or perhaps all of the scripts

Matthew W. Brown, Ph.D. • 662.325.2406 • mwb250@msstate.edu • 007 Harned Hall
http://amoeba.msstate.edu

will find even wider use.

Thank you for your thorough review, constructive criticism, and positivity about the potential of our work.

Reviewer #3:

PhyloFisher

Phylofisher is a set of scripts, database and tools to generate standardised phylogenies to try and resolve interesting or unknown phylogenetic relationships in the eToL.

The author's identify the need for these tools, as there are a multitude of published trees looking at similar datasets but note that they often show differening results (and interpretations) due to the differences in their curation and methods used. They go on to create a database and set of tools in order to help with this, and also test their tool on several contentious evolutionary relationships serving as a proxy to dtermine their tool use and pipeline decisions.

The author's built a curated datsaet of 240 proteins from 304 diverse taxa from the eToL ("PhyloFisher_Proteome_Data.tgz" and detailed philosophically from Ln171++) - however, this is not currently located as a download in the supplementary for reviewers, and on contacting the journal editor they did not have a copy either. This is not particularly useful. It is also not part of the conda install, and not available on their github. For publication this should be made available, open and placed somwhere like figshare or another repository, versioned and archived with a DOI. It is an important part of the pipeline (unless you create your own DB, which I feel is not adequately explained either), and without it the claims surrounding phylogenetic relationships further in the paper cannot be repeated or tested.

We would like to apologize for the lack of availability and confusion as to the differing nature of our database and the file “PhyloFisher_Proteome_Data.tgz”. Due to decisions during further testing, the file “PhyloFisher_Proteome_Data.tgz” no longer exists. All proteomes used as input to construct the provided starting database as well as the orthologs and paralogs that make up the provided starting database can be retrieved via wget from:

https://ir.library.msstate.edu/bitstream/handle/11668/19731/Tice_etal.PhyloFisherDatabase_v1.0_Apr.11.2021.tar.gz

After uncompressing the file, the input proteomes can be found in PhyloFisherDatabase_v1.0/database/proteomes, the set of orthologs in the starting database can be found in PhyloFisherDatabase_v1.0/database/orthologs, and the set of paralogs in the starting database can be found in PhyloFisherDatabase_v1.0/database/paralogs. Information for each taxon in the database as well as accession information for the input proteomes or nucleotide data used to predict the proteomes can accessed either by the script *explore_database.py* or by manually opening the file PhyloFisherDatabase_v1.0/database/metadata.tsv.

Also to be able to test the tool for review it should have been accessible. This seems to be somewhat of a missed opportunity and largely reflects the review decision in my opinion.

We apologize for not providing private access to reviewers to facilitate a more complete and thorough review process. We would like to explain that our decision to not have the tool publicly available until acceptance of our manuscript was one based on caution. We did not want researchers actively using tools within the package if during review a critical problem was brought to our attention regarding our strategy for ortholog collection or any of the functions of the utilities within PhyloFisher are capable of performing. However, all the code is now available via conda, pip, and github. We have also provided the PhyloFisher manual as a part of our resubmission which we believe will alleviate many concerns that arose regarding lack of transparency or documentation.

PLEASE NOTE: We have provided the Manual as a supplemental file in this submission. However, it is an ever-evolving document, we ask that you please retrieve the most up-to-date version at http://amoeba.msstate.edu/share/PhyloFisher_Manual.pdf.

Additionally, we request that if any coding issue comes up, please first "conda update phylofisher" (or PIP). If this does not resolve the issue then please email us, which may be done confidentially through the *PLoS Biology* editor.

The tools/scripts themselves are available at github, and can also be installed with modern package manager tools such as conda and pip, which is most important for a modern tool that encompasses and relies on many dependenicies - so, that is good to see. Installation for this review used miniconda on ubuntu linux, which seemed to work fine. There are a few issues with some scripts, which are noted below…

The methodology of the reconstruction of the eToL and others is quite well explained but is somewhat burried in the supplementary (ln215++), it would be good to see this in a more user friendly format on the GitHub repo (e.g. as a wiki or similar) as currently there is rather little on the functioning of the scripts located there. Also a walkthrough and some example datasets of the best use cases would be extremely valuable for users who want to learn and get started quickly. A tool that is advertised as "easy-to-use" really needs something like this accompanying it. Ln 69 mentiones a "manual", but where is this? There are a great many accessory scripts included in this tool and they should be mentioned and explained with reference to their use, expected inputs and outputs. e.g. Fig 1. Although a large image with a lot of information displayed, many of the boxes/panels are represented by scripts within the phylofisher package, therfore it would be quite useful for the names of those scripts to appear in the boxes that represent them as a hint for potential users.

Further, Ln90 talks about an "input metadata file" but this file and the format it expects are not detailed anywhere (what inputs are required, what format should they take). A version is included in the github repo but refers to hard coded details not present in the repo itself or values are left as "XX" without reference to what values they could take, so the example is not particularly useful as a starting point. There does not have to be a complete walkthough of how to assemble the same dataset the author's produced for the paper, although that would be excellent and reflect

the open nature and quality of PloS (the genomes/transcriptomes are detailed in supplementary but perhaps they should be offered as a static tarball of assemblies etc), but a smaller and slightly contrived example with a small set of easily accessible Euk genomes would suffice - this should detail the full process, all commands needed, even those outside of the PhyloFisher package to show what is required to start...

Similarly, a walkthrough on the steps to create a custome DB (if not using the one referenced in the paper) would be extremely valuable, there is some barebones of this on their github already but it is not overly clear on any prior steps, and it is also unfinished...

We are regretful for this apparent lack of documentation and transparency. We have provided the PhyloFisher manual as part of our resubmission. In it, we provide sections detailing installation, complete usage information for all scripts including expected input and output, and a detailed example walkthrough of the main workflow.

PLEASE NOTE: We have provided the Manual as a supplemental file in this submission. However, it is an ever-evolving document, we ask that you please retrieve the most up-to-date version at http://amoeba.msstate.edu/share/PhyloFisher_Manual.pdf.

Running "forge.py" and "fisher.py" without any arguments throws a python error, they should at least display the help menu instead.

All python scripts that have required arguments now print a "usage" menu when they are run without any arguments. All scripts have a -h/--help option to display a full help menu with details of the available options. We hope the documentation outlined in our provided manual clarifies this.

The paper also mentions a tool "ParaSorter" (e.g. Ln108) which also appears in the supplementary, but I do not see it as an installation candidate or any code within the group's github anywhere. Or at least it is non-obvious to me. It sounds cool! So, where is it? Also, I think it probably deserves more than one runaway sentence...as it could be a useful tool outside of PhyloFisher in-and-of itself. It too should be made available as part of the review and eventual publication/repository. Apologies if I have missed it, but I went through the code where I think it should be and there's nothing there.

ParaSorter is included in all installs of PhyloFisher and available as an application from the github. If installing via conda, once the install is complete and the environment has been activated simply type "parasorter" to open the application.

While we have tried to make many of the tools provided in PhyloFisher as "stand-alone" as possible, as we explain in our response to Reviewer 2's inquiry about this, we are disappointed to say that ParaSorter's functionality is reliant on the PhyloFisher framework and cannot be used outside of it unfortunately.

Experimentally, the methodologies employed to produce an alignment and the order of the specific

tools used to then produce a phylogeny is sound and I have no concern over the methodology here. The selection of trimming tool is nicely corroborated with the use of RTC scores for genes trees per different settings across trimming programs. Indeed, many tools are tested in this manner throughout the script. Nonetheless, I think that it would help, e.g. those coming to a tool advertised as "easy to use", if the choices could have a little more of the author's thought processes/philosophy given as to why those tools, and in that order, have been chosen. Whilst I am very familiar with these tools, as are obviously the author's, if they would like their suite of tools to be adopted as a standard protocol - or at least a starting point - it might help their case to walk through the more novice users with their design choices in a more friendly format.

We did not perform the necessary experiments to show the optimum combination of a factorial number of possible parameters across 4 tools used in sgt_constuctor.py on sequence files prior to tree construction via RAxML. We employ multiple state-of-the-art strategies in an effort to reduce MSA inaccuracy which naturally leads to inaccuracy in the resulting phylogenies. The order that each tool is applied to the sequence files is inherent to the nature of the function of each tool. We have provided *sgt_constructor.py* as a means for less experienced users to filter, align, trim and construct phylogenetic trees in a rigorous fashion from homologs in a single simple step. However, we realize as research in this field continues optimum combinations of these program's and their parameters may be discovered and PhyloFisher allows users the flexibility to alter the provided parameters to account for this.

Whilst I don't think the issues with the documentation, or lack of walktrhough examples, and missing dataset detract from the paper or the tool in and of itself, for a tool that is described as "easy to use" and aimed at including more researchers being able to produce better phylogenies (and so better test their hypotheses) via a standardised pipeline (especially those who may not have much experience in determining what they need to start with) I would expect there to be much better documentation, and for the database to be accessible immediately. The merit of the paper and the security of it rests on it being tested externally before publication. Not least for it to be considered to be published in a PloS journal.

Thank you for your thorough and critical review. We hope that PhyloFisher helps to increase reproducibility and transparency in phylogenomics and that the provided manual documenting PhyloFisher along with our other responses are thoroughly convincing of our commitment to these objectives.

PLEASE NOTE: We have provided the Manual as a supplemental file in this submission. However, it is an ever-evolving document, we ask that you please retrieve the most up-to-date version at http://amoeba.msstate.edu/share/PhyloFisher_Manual.pdf.

Minor corrections to Text:
Ln109 "base" should be "based"
Corrected.

Matthew W. Brown, Ph.D. • 662.325.2406 • mwb250@msstate.edu • 007 Harned Hall
http://amoeba.msstate.edu

Ln189 insert "a" between "by" and "few"
Corrected.

Ln190 remove "more" between "much" and "widely"
We kindly disagree.

Ln238 insert "that" between "analyses" and "are"
We kindly disagree.

Supplementary
Ln145 script name is missing an "s"
Corrected.

Ln149 "choose" should be "chose"
Corrected.
Ln162/165 script name is incorrect
Corrected.

Ln 173 "the taxa" should be "each taxa"
This has been changed to "each taxon".

Ln175 "culled" should be "retrieved"
Corrected.

Ln179 comma after "taxa"
Corrected.

Ln186 mentions inaccessible file
This file no longer exists. All proteomes used to as input for PhyloFisher during database construction can now be obtained via wget from:

https://ir.library.msstate.edu/bitstream/handle/11668/19731/Tice_etal.PhyloFisherDatabase_v1.0_Apr.11.2021.tar.gz

After uncompressing the file, the input proteomes can be found in PhyloFisherDatabase_v1.0/database/proteomes. Information for each taxon in the database as well as accession information for the input proteomes can accessed either by the script *explore_database.py* or by manually opening the file PhyloFisherDatabase_v1.0/database/metadata.tsv.

Ln193 "organisms"
Corrected.

Ln200 - where is this tool ParaSorter?!

ParaSorter is included in all installs of PhyloFisher and available as an application from the github. If installing via conda using the step by step instructions provided in the manual, simply type "parasorter" into the command line to open the application once the install is complete and the environment has been activated. Windows users must retrieve ParaSorter from the github.

Ln202 - not shipped?!
The database that is made up of sequences demarcated as either an ortholog or paralog, the profile HMMs corresponding to each ortholog resulting from our manual curation effort, and the input proteomes used to create the database are available for download via wget from:

https://ir.library.msstate.edu/bitstream/handle/11668/19731/Tice_etal.PhyloFisherDatabase_v1.0_Apr.11.2021.tar.gz

Ln211 - this should be subset_tools.py
This script no longer exists. The functionality of this script has been divided into three scripts (*select_orthologs.py, select_taxa.py*, and *prep_final_dataset.py*) to make the subsetting process more intuitive. This decision is based on our personal experience with the workflow and beta tester recommendations. The script name no longer appears in the text and the github has been updated accordingly.

Ln217 - "no_gaps_stops.py" doesn't exist?
This script no longer exists. The code now exists as a function (delete_star_gaps) found in the scripts *sgt_constructor.py* and *matrix_constructor.py* (formally *forge.py*). Any mention of the script has been removed from the text.

Ln387+388+412 - script name is wrong compared to github
This script is now called *fast_site_remover.py*. The name has been updated throughout the text and the github.

Ln432/3 program name incorrect
This script is now called *random_resampler.py*. The name has been updated throughout the text and the github.

Ln560 program name incorrect
This script is now called *fast_site_remover.py*. The name has been updated throughout the text and the github.

References

Shen XX, Opulente DA, Kominek J, Zhou X, Steenwyk JL, Buh KV, Haase MAB, Wisecaver JH, Wang M, Doering DT, Boudouris JT, Schneider RM, Langdon QK, Ohkuma M, Endoh R, Takashima M, Manabe RI, Čadež N, Libkind D, Rosa CA, DeVirgilio J, Hulfachor AB, Groenewald M, Kurtzman CP, Hittinger CT, Rokas A. Tempo and Mode of Genome Evolution in the Budding Yeast Subphylum. *Cell*. 2018 Nov 29;175(6):1533-1545.e20. doi: 10.1016/j.cell.2018.10.023. Epub 2018 Nov 8. PMID: 30415838; PMCID: PMC6291210.

Salomaki ED, Terpis KX, Rueckert S, Kotyk M, Varadínová ZK, Čepička I, Lane CE, Kolisko M. Gregarine single-cell transcriptomics reveals differential mitochondrial remodeling and adaptation in apicomplexans. *BMC Biol* 19, 77 (2021). https://doi.org/10.1186/s12915-021-01007-2
