# Supplementary material for: PhyloFisher: A phylogenomic package for resolving eukaryotic relationships
Source: PLoS Biol. 2021 Aug 6;19(8):e3001365. doi: 10.1371/journal.pbio.3001365 (PMC8345874; doi:10.1371/journal.pbio.3001365)
Supplement: Attachment — Submitted filename: Tice_etal.PhyloFisher.Response.to.Editor_Reviewers.pdf [file pbio.3001365.s004.pdf]

Dear Matt,

Thank you for submitting your revised Methods and Resources paper entitled "PhyloFisher: A phylogenomic package for resolving eukaryotic relationships" for publication in PLOS Biology. I've now obtained advice from the original reviewers and have discussed their comments with the Academic Editor.

Based on the reviews, we will probably accept this manuscript for publication, provided you satisfactorily address the following data and other policy-related requests.

IMPORTANT:

a) Our IThenticate software detects a significant similarity between lines 418-427 and a BMC Biology paper (https://secure-web.cisco.com/19LF_Q036R5ev04buArooVqNrqZo7OR6P4i4h-d67FLopsyJ6b9s8khC8aOvi0Rq-4KFcDOBOgR2w1xbxwQNXDq6gWHqTItyu81htudYeWlNsBVf-UUN49yOqLhucaZjnwL4uWx1Cb-sm2DmqzRSySIFjT_s2OKctWe4V4OwNQDV4zNznbTsqOgIVTk3q1CTrPgWtInoWGiU0i9JY1wsuEWwrsOJOPeYNpeUTGgFU9Xb_8yhY6K43jRyppA3Ezz4gCV7EIJYKYjwXQPdou_Bi_sQuohDPtGgLahsUfmVO937bhoOBlZJSEtGhDnx7GIeR/https%3A%2F%2Fbmcbiol.biomedcentral.com%2Farticles%2F10.1186%2Fs12915-021-01007-2) by a subset of your co-authors. Please could you adjust the wording of this section to minimise any copyright issues?

Sorry about this oversight, you are correct. We tried to state the exact way in which this data was handled, and the statement is close to the BMC paper listed. We have revised the manuscript to read “The resulting homolog trees were examined manually with the included tool “ParaSorter” to select orthologous sequences, demarcate paralogs, and remove contaminating sequences using the logic outlined in the section “Phylogenomic dataset construction and analyses” of [8].”

b) Please could you attend to my Data Policy requests further down. Essentially, while it may be that some of your Figs can be generates using the software package and the database provided, it's not clear whether this applies to all of the Fig panels (especially the supplementary Figs S2, S3, S4, S5, S6, S7, S8, S10, S12, S13, S14). In each case, please can you supply the underlying data and cite its location clearly in the respective Fig legends? If the data can be generated using the Phylofisher software and the dataset then please specify this (including URLs) in each legend.

We have now provided all data associated with each figure. These data along with the R code, where necessary, is also available in the supplement associated with each figure, if the figure was produced in R. For each figure the statement reads “Data associated with this figure is available in the directory archive SXFig.tgz within the data archive available from

Matthew W. Brown, Ph.D. • 662.325.2406 • mwb250@msstate.edu • 007 Harned Hall
http://amoeba.msstate.edu

https://ir.library.msstate.edu/bitstream/handle/11668/19731/Tice_etal.PhyloFisher.DATA.tgz.” (X is changed per figure).

As you address these items, please take this last chance to review your reference list to ensure that it is complete and correct. If you have cited papers that have been retracted, please include the rationale for doing so in the manuscript text, or remove these references and replace them with relevant current references. Any changes to the reference list should be mentioned in the cover letter that accompanies your revised manuscript.

We expect to receive your revised manuscript within two weeks.

To submit your revision, please go to https://secure-web.cisco.com/1VAmShMRg2iu9DInxVJL6V6wCjL3izNcMzTuA3HYEO_v9tdZSY91su9EDUpEZOwhwWOfRuyjuGEyZTTqkoNqPsDzByVaCyRQUTUpYXyVk-zAVDhmcFx3NBaGW0R6hEUK0OECQyUiHfdRC6m6CI9UBzAPE48S_rlliPhbX-30_l3w_OugLUxkQqRBOhk9RDlSKHlkvLukwJbf3jZ5w96P2MkIot6eFkQ3LRY6-rTkmKGJ32Q5Irx3OvzXJV6cKu5LVmyfBo1RmwoI_BGHyaE8BrLahsfBmPRImyEpVvE0P7qEcwNNA2bxF402iorljR1Nc/https%3A%2F%2Fwww.editorialmanager.com%2Fpbiology%2F and log in as an Author. Click the link labelled 'Submissions Needing Revision' to find your submission record. Your revised submission must include the following:

- a cover letter that should detail your responses to any editorial requests, if applicable, and whether changes have been made to the reference list

We have no editorial requests.

- a Response to Reviewers file that provides a detailed response to the reviewers' comments (if applicable)

No responses are necessary.

- a track-changes file indicating any changes that you have made to the manuscript.

A track changes file has been included in the submission.

NOTE: If Supporting Information files are included with your article, note that these are not copyedited and will be published as they are submitted. Please ensure that these files are legible and of high quality (at least 300 dpi) in an easily accessible file format. For this reason, please be aware that any references listed in an SI file will not be indexed. For more information, see our Supporting Information guidelines:
https://secure-web.cisco.com/1vwbXYwEg6qwB1Eft5Fg19kZAkWC_U6P6kcZ2QtxXq6v6T6bZlApC8TntIdeOhVXg_VLYQ85eUUGzwe30P0cAJQUrSOt2P-8PLshZA-6DWCnT-vvwAUWwoiOAcdeNfdDFyB7EMaOznQKPHMl0J-

U1BJIJ4ZDl1equMDaIOs2VPcZJdqTqJkUYD8cULuz7qoLA79qJMaQlx3lEEwx6BcM97H__si
-6HoOBrK5ejy5kt5dFT2BGJNXTSgft0vNxUT2cjZ3UFTRgajsVDQRtPU-
sKVx7gUnkcZMbyaAQ1UKhmMITsV5Q_pwQg5vf1xeXrTkN/https%3A%2F%2Fjournals.plo
s.org%2Fplosbiology%2Fs%2Fsupporting-information

Published Peer Review History
Please note that you may have the opportunity to make the peer review history publicly available. The record will include editor decision letters (with reviews) and your responses to reviewer comments. If eligible, we will contact you to opt in or out. Please see here for more details:
https://secure-
web.cisco.com/1OVlkWDVZmIKzlIqUqC4bJYJErvvYMKHtHb702lmSjiGwEHP1yMgcnaUyr
WKVVFp6V2Drs2mq1L99J1GnvM1aN6dOp31h7h8zHN6Ge4hYOgOMHDFNcc9yWXPzllPf6
vXr3UxC-P1hSbDmXXMI6D8ziR0JnB6WCSvTwpGE-
edbQlAfc4eybNZJPZynAl8BC1w6OB2d4G0bnS44yuWZ7_xUUmQkYd1DgpIDyVyfEd8WeA
qnTtEokUc6_AN9h6Ksq5leM3_O99A9wkMP333PRCzUmOn7QUpqWx9DSaSGssFTIrHu3H
GhBC2fsd_GqBbTa6NC/https%3A%2F%2Fblogs.plos.org%2Fplos%2F2019%2F05%2Fplos-
journals-now-open-for-published-peer-review%2F

Early Version
Please note that an uncorrected proof of your manuscript will be published online ahead of the final version, unless you opted out when submitting your manuscript. If, for any reason, you do not want an earlier version of your manuscript published online, uncheck the box. Should you, your institution's press office or the journal office choose to press release your paper, you will automatically be opted out of early publication. We ask that you notify us as soon as possible if you or your institution is planning to press release the article.

Protocols deposition
To enhance the reproducibility of your results, we recommend that if applicable you deposit your laboratory protocols in protocols.io, where a protocol can be assigned its own identifier (DOI) such that it can be cited independently in the future. Additionally, PLOS ONE offers an option for publishing peer-reviewed Lab Protocol articles, which describe protocols hosted on protocols.io. Read more information on sharing protocols at https://secure-
web.cisco.com/12NbmfLCcKUxoGJ5WTJg6EDA7A-OAn-
g_1xMcMeNkQ5gotXA16K4phmFNceZF3P6MaYZmPQdWbuCKwTEThATLLn2pqS4DXCY
5gqFfdCtzuJXq4-vjfeptCMcnulzBylRCEGiclFpnTeMLcUhgQP-
s14ZU2jNkbQ1SuMLq_BNcc8FoSDpE25CMx54zs1Ks4yDc5f2LuD0JamiU3dMiB4W8M-
SIRBx3U3ajm5BItjfwvok3vrD6rYdZ6w79jQvsjlQWS_tsP7Yr_pXEWiQ1F5zk69LuE_CuZAf
mgGDXD-
2vV9bvlfcBkUCG01e8CC6J2PLe/https%3A%2F%2Fplos.org%2Fprotocols%3Futm_medium%
3Deditorial-email%26utm_source%3Dauthorletters%26utm_campaign%3Dprotocols

Please do not hesitate to contact me should you have any questions.

Sincerely,

Roli

Roland G Roberts, PhD,
Senior Editor,
rroberts@plos.org,
PLOS Biology

DATA POLICY:

You may be aware of the PLOS Data Policy, which requires that all data be made available without restriction: http://secure-web.cisco.com/1ottBSmXspZV3es3QnFyre6mNUWFUwy7Td57Pf8GTdiwYoeJinGR40einYTM-uK16IV8kXF4-pt2bo9Db6QbNLDIY8BBZd2-UCvNbAb5SEQVsEyFtdEy1P1-kPDNNSIY8zr3ZOGVSUv8yX92XW6-QhwFsdkImflBgBX0IGADBLzQR7rMN85Uxd3t-NcDEmWB2KoW_jZmWYYJ494W7ya9AtufsR0BAsUT_8JStGO7vtmzFTHzlPnmktpksS0axkAbiKBc7cMJJQ5rd0VQ2s4rEYRAZ3LKW5nOyL8PQm6oIJcoTso-66y01rfNijOEGS3cT/http%3A%2F%2Fjournals.plos.org%2Fplosbiology%2Fs%2Fdata-availability For more information, please also see this editorial: http://secure-web.cisco.com/1jA_AZvycLHpdP9clyo4KDwjODO9ZzBMzhyrVewlzRyl_GrjvhmKw2ygZ-v42WHseL8gmy6gEd7ja-LXWodBhN0DZACItWQRKu5gJwe704ANHAcoOeRUTi32vVS0uN4ioGedmnD9f4rU-1Ov0Mc9xOHyEbSyAf9GogQwALh-yVMnm9uzh61rq8eumRvXZ2BsXF4s1tjKnzBlxiigMkghhCLeZ5fqv4gDCURbhT89WETtePA4BeAKRTZ68ZUPCOXbIGkDvz_1p0Of1x1ImgiwL-KpvFLlYCAnLZWGf9vkGnTvXeh7piT7EgL7DoP-ZZ_3I/http%3A%2F%2Fdx.doi.org%2F10.1371%2Fjournal.pbio.1001797

Note that we do not require all raw data. Rather, we ask that all individual quantitative observations that underlie the data summarized in the figures and results of your paper be made available in one of the following forms:

1) Supplementary files (e.g., excel). Please ensure that all data files are uploaded as 'Supporting Information' and are invariably referred to (in the manuscript, figure legends, and the Description field when uploading your files) using the following format verbatim: S1 Data, S2 Data, etc. Multiple panels of a single or even several figures can be included as multiple sheets in one excel file that is saved using exactly the following convention: S1_Data.xlsx (using an underscore).

2) Deposition in a publicly available repository. Please also provide the accession code or a reviewer link so that we may view your data before publication.

Regardless of the method selected, please ensure that you provide the individual numerical values that underlie the summary data displayed in the following figure panels as they are essential for readers to assess your analysis and to reproduce it: Figs S2, S3, S4, S5, S6, S7, S8, S10, S12, S13, S14.

NOTE: the numerical data provided should include all replicates AND the way in which the plotted mean and errors were derived (it should not present only the mean/average values).

IMPORTANT: Please also ensure that figure legends in your manuscript include information on where the underlying data can be found, and ensure your supplemental data file/s has a legend. If the data can be generated using the Phylofisher software and the dataset then please specify this (including URLs) in each legend.

Please ensure that your Data Statement in the submission system accurately describes where your data can be found.

DATA NOT SHOWN?

- Please note that per journal policy, we do not allow the mention of "data not shown", "personal communication", "manuscript in preparation" or other references to data that is not publicly available or contained within this manuscript. Please either remove mention of these data or provide figures presenting the results and the data underlying the figure(s).

We have combed through the manuscript, and we are unable to find any instances of data not shown. All data is shown.

REVIEWERS' COMMENTS:

No response is necessary to these reviewer comments.

Reviewer #1:

The authors have added an additional analysis of another area of the tree of life. This is adding to the applicability of the software. The authors have furthermore addressed most of the comments of the reviewers. The addition of more authors is justified.

Reviewer #2:

They have sufficiently addressed most of the comments and made changes that have strengthened the manuscript. While I don't necessarily agree with all of their responses, it's not imperative that I agree with everything for this to be publishable. This is a good contribution to the community who works on resolving deep relationships in the eukaryotic tree of life.

Reviewer #3:

Initially I was worried that the paper and accompanying tool did not do enough to allow users to get started easily, however this has been massively improved on re-submission. There is a now a very well documented manual that accompanies the set of scripts/programs outlined in the paper. The tools themselves have also been improved and the starting database is now available. These inclusions and updates remove any doubts that it will not be a useful tool for the community. Therefore I am happy to recommend the manuscript's acceptance and the author's replies subsequently I have no further recommendations and am looking forward to using the software in some analyses.

____

In compliance with data protection regulations, you may request that we remove your personal registration details at any time. (Use the following URL: https://secure-web.cisco.com/1jfWg40xHzHl1d30YrZnF-qWmc_a4nEV0xsLsDtBX0gD_PqSKM2ciJrP_0vA_CH32FmvGein0JIa-8gkXF_qf4_nMq3EWZPDuvliTa9hJCu5id3ZeDpsrCVw4tLpPVXAvzxZdubnh8cOyOrSosqtTqUACi9BhcpbchJQJcCXJs0CnFfncjBKNlg8kzv1MeGggkQ08MHA8Lage31RZb-34e5AZPdOMiiQ29f8fM7a8J5e39gRCNUSL5hDRwIySdirPQkEyNzOJRG5yWlbmZfo1uTpc4ar_gJpXB2I_oMWKa-Ql7xwPBCDbIEZShSw3tt0E/https%3A%2F%2Fwww.editorialmanager.com%2Fpbiology%2Flogin.asp%3Fa%3Dr). Please contact the publication office if you have any questions.
